# Ferritin‐Doped Nanoparticles Triggered Tumor‐Specific Darkening for Enhanced Photothermal Tumor Ablation and Immune Activation

**DOI:** 10.1002/adhm.202505119

**Published:** 2026-01-24

**Authors:** Haidong Zha, Xiao Liang, Long Xi, Jiamao Chen, Wenzhao Zhong, Zhen Yuan, Meng Xu, Ying Zheng

**Affiliations:** ^1^ State Key Laboratory of Mechanism and Quality of Chinese Medicine Institute of Chinese Medical Sciences University of Macau Macau China; ^2^ Guangdong‐Hong Kong‐Macao Joint Laboratory for New Drug Screening University of Macau Taipa Macau China; ^3^ Faculty of Health Sciences University of Macau Macau China; ^4^ School of Pharmaceutical Sciences Guangzhou University of Chinese Medicine Guangzhou China

**Keywords:** fenton reaction, human ferritin heavy chain, photothermal therapy, transferrin receptor 1, vascular disrupting agent

## Abstract

Combining the lysosomal targeting ability of human ferritin heavy chain (HFn) and the strong oxidizing property of gallic acid‐iron complex (GA‐Fe), a hybrid nanoparticle HFn/GA‐Fe was synthesized by a cross‐linking reaction. We discovered that HFn/GA‐Fe would specifically darken the color of tumors after intravenous injection without rendering notable toxicity in normal organs, as observed by visual inspection and photoacoustic imaging. HFn/GA‐Fe specifically bound to HFn receptor (TfR1/TIM‐2) and aggregated at the lysosomal pH. HFn/GA‐Fe induced endothelial cell death and tumor‐specific hemorrhage by generating reactive oxygen species (ROS). Under laser irradiation, the leaked deoxyhemoglobin significantly enhanced the photothermal effect and subsequently triggered anti‐tumor immune responses. The cavities of ferritin nanocages can further encapsulate drugs, endowing them with broader application prospects.

## Graphical abstract

Ferritin‐doped hybrid nanoparticles triggered accurate tumor‐specific hemorrhage for enhanced in situ photothermal therapy and evoked anti‐tumor immunity. Notably, HFn/GA‐Fe specifically induced tumor hemorrhage 12 h after intravenous injection, resulting in visible color darkening. Through photoacoustic imaging, tumor vessels could be clearly observed as damaged.

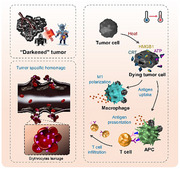

## 1 Introduction

Breast cancer has surpassed lung cancer to become the most prevalent cancer worldwide in 2020, and the incidence rate is still on the rise [1]. Recent research has applied many therapeutic strategies, such as chemotherapy, radiotherapy, and immunotherapy to treat cancer [2, 3, 4]. However, the rapid progression of tumors in clinical practice is still hard to control. Tumor induces angiogenesis to ensure nutrition and oxygen supply. Without angiogenesis, tumors can only grow up to around 1–2 mm^3^ [5]. Hence, targeted destruction of tumor blood vessels is a potential method to effectively inhibit cancer progression [6]. Currently, the strategies to target tumor blood vessels include inhibition of vascular endothelial proliferation [7], tumor vascular infarction [8], and tumor vascular disruption [9]. Damage to the existing tumor blood vessels exhibits strong synergistic effects with chemotherapy and photothermal therapy (PTT) [10, 11]. Some vascular‐disrupting agents (VDAs) have entered clinical trials, such as combretastatin A4, Flavone‐8‐acetic acid (FAA), and 5,6‐dimethylxanthone acetic acid (DMXAA) [12]. However, most of these VDAs are highly toxic and have failed in subsequent clinical trials [13, 14]. Thus, the development of novel VDAs with precise targeting and low toxicity characteristics is urgent to facilitate the clinical transformation of tumor‐vessel‐targeting therapy.

Human ferritin is a protein nanocage primarily involved in the storage and transport of iron within cells [15]. The human ferritin heavy chain (HFn) targets tumors by recognizing highly expressed transferrin receptor 1 (TfR1) in humans and T‐cell immunoglobulin and mucin domain 2 (TIM‐2) in mice [16, 17]. TfR1 mediates cellular iron uptake and is lowly expressed levels in most normal human cells. Tumor cells require high levels of iron for rapid proliferation, and thus TfR1 expression is significantly upregulated [18]. TIM‐2 is a gene involved in immune regulation, and its expression on 4T1 cells was also verified [19]. HFn was found to localize and aggregate in lysosomes after internalization [20, 21]. Besides, the nanocage structure of HFn disassembles at extreme acid/alkaline conditions (e.g., pH = 2.0/pH = 11.0) and reassembles at neutral pH, thus functioning as both a targeting ligand and a drug carrier [22].

The rapid chelation between metal ions and phenolic ligands enables the formation of uniform metal‐phenolic network (MPN) coatings on various substrates (e.g., biological entities, organic materials, and inorganic materials) within minutes under aqueous conditions [23]. The gallic acid‐iron (GA‐Fe) complex was first used as iron‐gall ink during the Renaissance, and its strong oxidizing properties damaged many famous masterpieces [24]. With this characteristic, GA‐Fe was employed for anti‐tumor chemodynamic therapy (CDT). CDT utilizes endogenous hydrogen peroxide (H_2_O_2_) in tumors to activate the Fenton reaction or Fenton‐like reaction, generating highly cytotoxic ROS to damage cancer cells [25, 26]. As a novel green therapeutic strategy, it has the advantages of high safety, good selectivity, and no limitation to tissue penetration depth [27, 28]. Besides, the broad near‐infrared (NIR) absorbance also makes it a promising photothermal agent in cancer therapy [29, 30]. Carriers such as liposomes [31], bovine serum albumin (BSA) [32], and mesoporous silica nanoparticles (MSN) have been used to deliver GA‐Fe to the tumor site [24]. The efficient and precise delivery of GA‐Fe is the key to achieving effective CDT.

**SCHEME 1:**
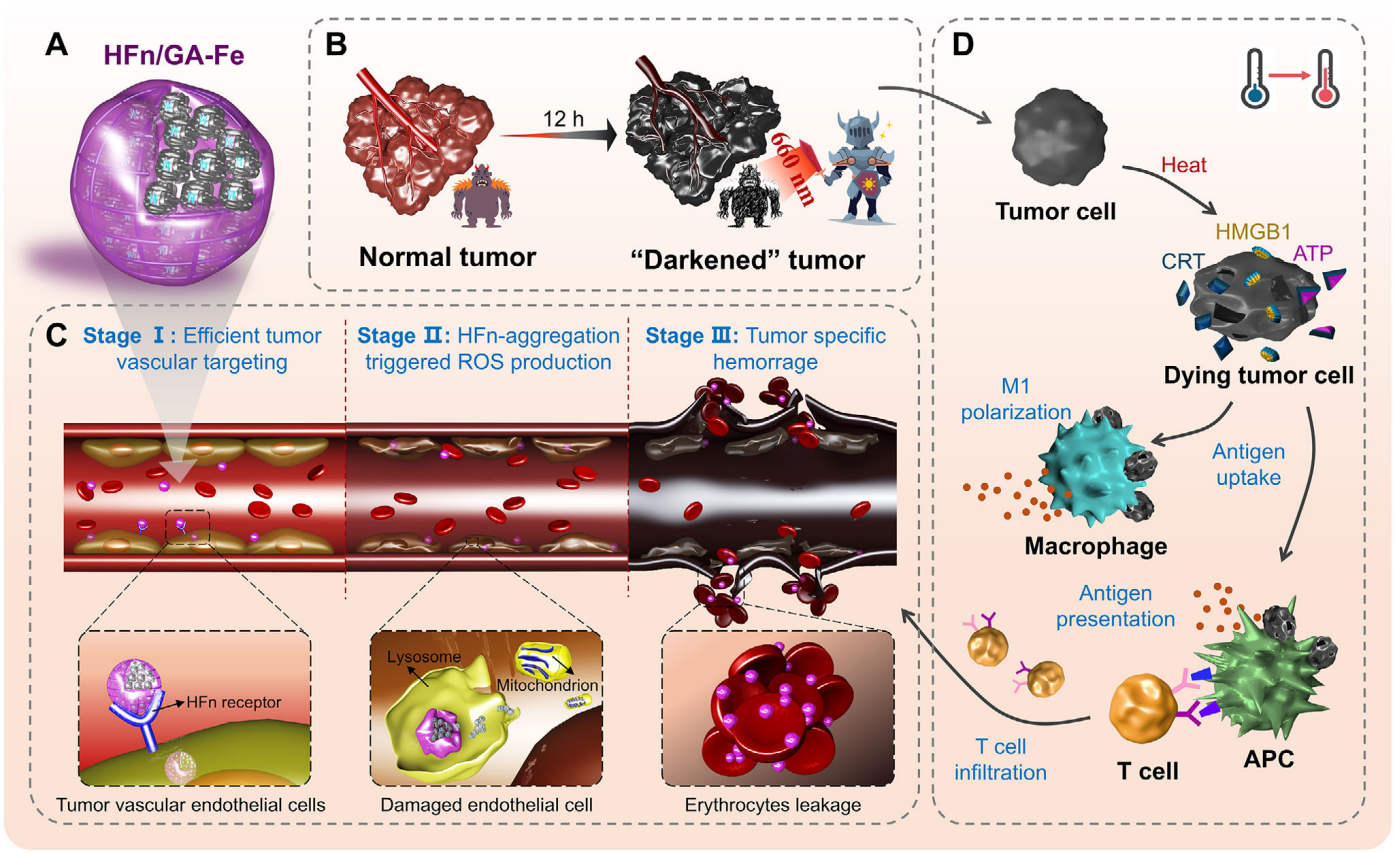
Schematic illustration of ferritin‐based tumor vascular disrupting strategy. (A) Hybrid nanoparticle HFn/GA‐Fe was synthesized through crosslinking HFn with a gallic acid‐iron complex. (B) After intravenous injection of HFn/GA‐Fe for 12 h, the tumor turned into a darkened color with strong near‐infrared (NIR) absorbance at 660 nm. (C) HFn/GA‐Fe specifically targeted the tumor site through HFn receptor binding. HFn/GA‐Fe aggregated in the lysosome, inducing ROS production and causing mitochondrial crista disorder. The death of endothelial cells at the tumor site led to hemoglobin leakage. (D) In situ PTT triggered immunogenetic cell death of cancer cells and evoked systemic anti‐tumor immunity.

In this study, we combined the precise targeting of HFn with the intracellular Fenton reaction property of the GA‐Fe complex via a simple cross‐linking reaction (Scheme 1). Surprisingly, the formed hybrid HFn/GA‐Fe nanoparticles rapidly induced tumor darkening at 12 h after intravenous injection, and no specific hemorrhage was observed in normal organs. We confirmed that the destruction of tumor vascular endothelial cells was triggered by lysosomal ferritin aggregation and ROS production by internalized HFn/GA‐Fe. Meanwhile, the leaked erythrocytes can absorb near‐infrared (NIR) light to further enhance PTT, thereby evoking anti‐tumor immune responses in vivo. In addition, the model chemotherapeutic drug doxorubicin (Dox) could be further encapsulated into the ferritin nanocage cavity for combination therapy. In this study, we developed a novel low‐toxic VDA to achieve precise targeting and treatment of tumors.

## 2 Result and Discussion

### 2.1 The Synthesis and Characterization of Hybrid HFn/GA‐Fe

**FIGURE 1:**
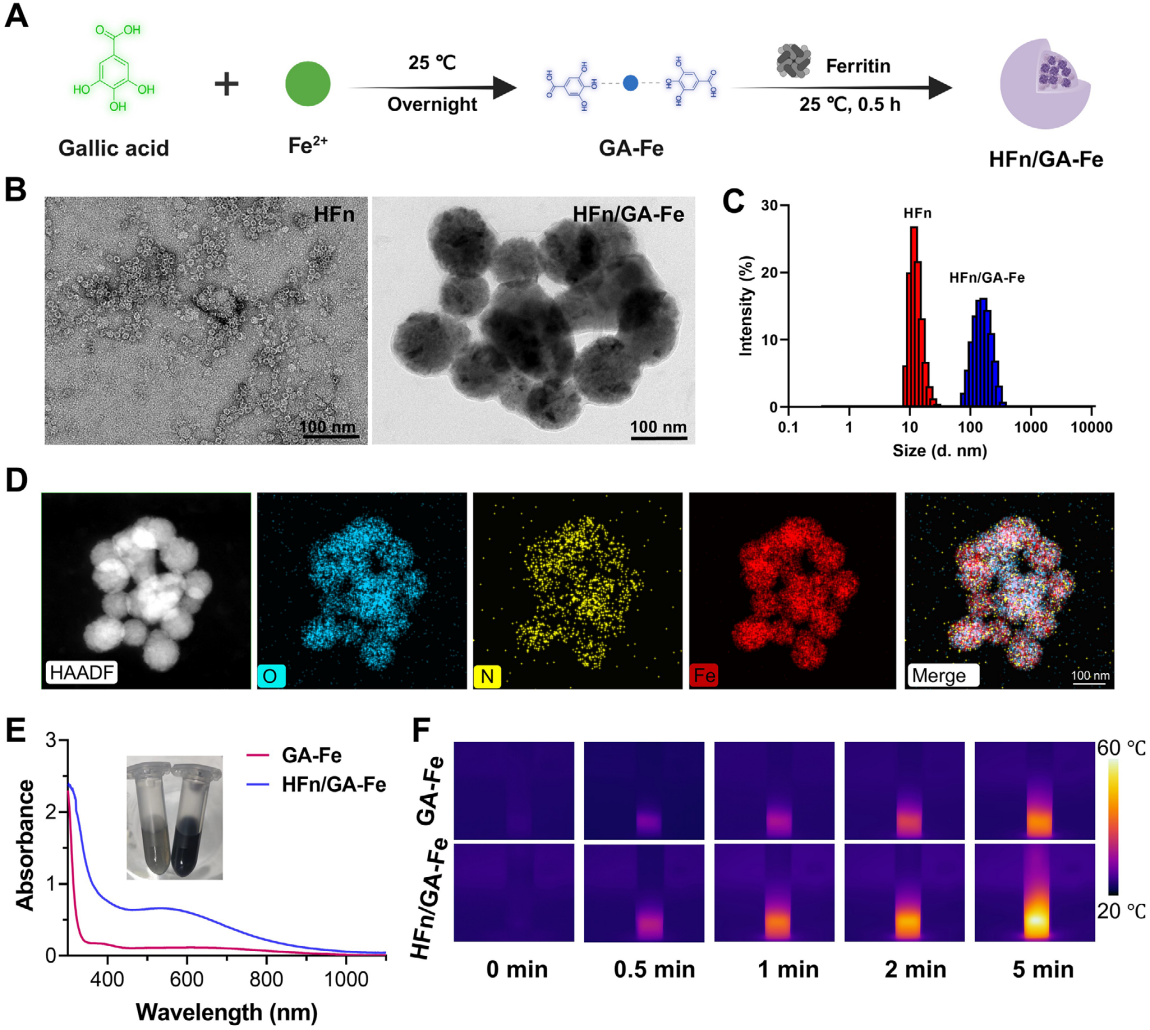
Preparation and characterization of HFn/GA‐Fe. (A) Schematic diagram showing the facile synthesis process of HFn/GA‐Fe. (B) TEM images of HFn and HFn/GA‐Fe. (C) Size distribution of HFn and HFn/GA‐Fe measured by DLS. (D) HAADF‐STEM image and O, N, Fe elemental mapping of HFn/GA‐Fe. (E) Representative photographs and wavelength absorption of GA‐Fe and HFn/GA‐Fe. (F) Thermal imaging of GA‐Fe and HFn/GA‐Fe under NIR laser irradiation at 0, 0.5, 1, 2, 5 min under 660 nm laser irradiation with a power of 1.5 W/cm^2^.

HFn was first recombinantly expressed by cloning the HFn‐encoding sequence into the PET30a plasmid, and its identity was confirmed by sequencing. The inserted fragment shared 100% identity to the human ferritin heavy chain gene retrieved from NCBI (Figure S1). HFn was successfully expressed and purified (Figure S2), and its molecular weight was consistent with theoretical predictions (Figure S3). After heating at 70°C for 10 min, the protein showed higher purity, which was attributed to the excellent thermal stability of HFn [33]. Subsequently, HFn/GA‐Fe hybrid nanoparticles were prepared by adding the GA‐Fe complex (Figure 1A). The purified HFn exhibited uniform size distribution and nanocage morphology in transmission electron microscopy (TEM) images (Figure 1B). When cross‐linked with GA‐Fe, it formed hybrid nanoparticles. The hydrodynamic diameter of HFn was 13.17 nm (PDI = 0.110) as measured by dynamic light scattering (DLS) [33], and that of HFn/GA‐Fe increased to 146.0 nm (PDI = 0.117) (Figure 1C). The interaction between GA and HFn was confirmed by protein fluorescent quenching assay. The fluorescence intensity of HFn decreased with the addition of gallic acid (Figure S4). Besides, HFn/GA‐Fe disassembled in PBS containing 1.5% Tween 20 (Figure S5), confirming the hydrophobic interaction between GA and HFn [34]. The elemental analysis by high‐angle annular dark‐field scanning transmission electron microscopy (HAADF‐STEM) further confirmed the existence of O, Fe, and N in HFn/GA‐Fe (Figure 1D). Compared to GA‐Fe, HFn/GA‐Fe exhibited higher NIR absorption and better photothermal conversion efficiency under 660 nm NIR laser irradiation (Figure 1E,F). The OD600 value was used as the index to more conveniently quantify the concentration of HFn/GA‐Fe (Figure S6). HFn/GA‐Fe exhibited good thermal stability after five rounds of thermal cycling (Figure S7). These results demonstrated that HFn is fully interacts with GA‐Fe to form a new nanoscale structure.

### 2.2 HFn/GA‐Fe Retained the Tumor Targeting Ability of HFn

**FIGURE 2:**
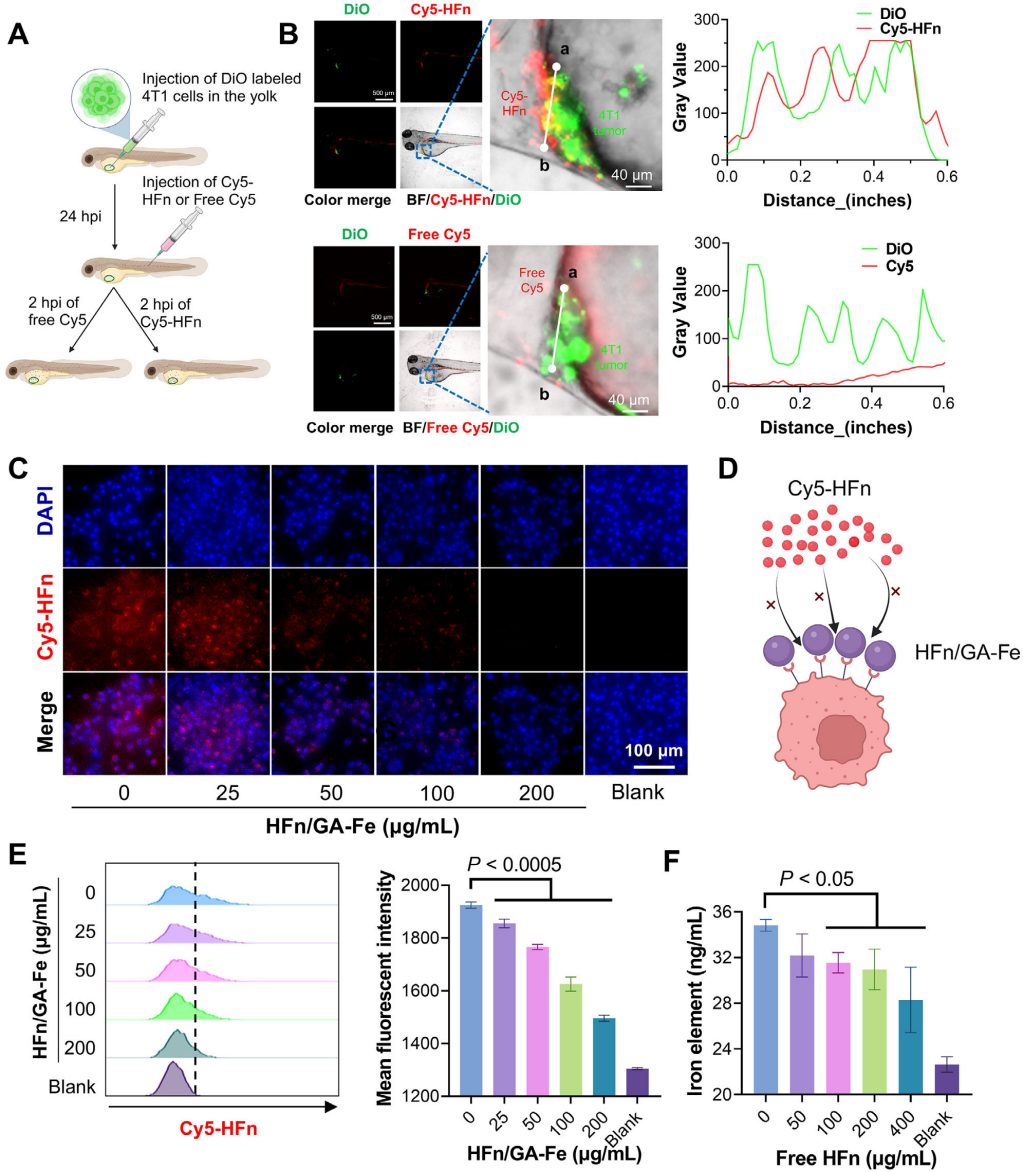
HFn‐mediated precise tumor targeting. (A) HFn targeting process in the zebrafish xenograft model. DiO‐labeled 4T1 cells were injected into the perivitelline space of wild‐type zebrafish embryos at 2‐day past fertilization. Cy5‐HFn and free Cy5 were intravenously injected. n > 15 samples per group. (B) Colocalization analysis of 4T1 cells and HFn in tumor‐bearing zebrafish. Fluorescence intensity traces of the white line from a to b were plotted. Green, DiO‐labeled 4T1; red, Cy5‐labeled HFn or Free Cy5. Scale bars: 500 µm; in amplified fields, 40 µm. (C) Cy5‐HFn (10 µg/mL) and HFn/GA‐Fe of different concentrations of 20, 50, 100, and 200 µg/mL were co‐incubated with 4T1 cells for 0.5 h and terminated with pre‐cooled PBS. The fluorescent images were pictured by DMI8 fluorescence microscope (Leica, Wetzlar, Germany). Scale bars: 100 µm. (D) Schematic diagram of the uptake inhibition assay. (E) Cy5‐HFn (10 µg/mL) and HFn/GA‐Fe of different concentrations of 20, 50, 100, and 200 µg/mL were co‐incubated with 4T1 cells for 0.5 h and terminated with pre‐cooled PBS. The cell fluorescent intensity was detected by flow‐cytometry (n = 3). (F) Free HFn was added at different final concentrations (0, 50, 100, 200, and 500 µg/mL) at 0.5 h before the supplement of 100 µg/mL HFn/GA‐Fe. After 1 h, the cells were treated referred to the last experiment and detected by iCAP Q ICP‐MASS (Thermal, America) (n = 3). Data are shown as mean ± standard deviation (SD). *P* stands for probability value used for hypothesis testing. All statistical analyses were performed using one‐way ANOVA.

Human ferritin can bind to TfR1 in humans or T‐cell immunoglobulin and mucin‐domain containing protein 2 (TIM‐2) in mice [17]. Zebrafish are emerging animal models for cancer research. Tumors developed in zebrafish are histologically and genetically to those in humans [35]. Transparent zebrafish allow visualization of the in vivo targeting process of nanomedicines [36]. We studied the targeting ability of HFn to 4T1 cancer cells using a zebrafish xenograft tumor model. Via microinjection, DiO‐labeled 4T1 cells (∼100 cells) were implanted into the perivitelline space around the yolk sac of zebrafish embryos. Subsequently, Cy5‐labeled HFn (Cy5‐HFn) or free Cy5 was injected into the dorsal vein to assess targeted binding during blood flow (Figure 2A). Compared with free Cy5, Cy5‐HFn showed significantly greater colocalization with the tumor cells (Figure 2B), which was consistent with the result of the mouse tumor model in previous studies [37]. To investigate the targeting ability of HFn/GA‐Fe to tumor cells, we compared the uptake of GA‐Fe and HFn/GA‐Fe by using intracellular iron content as an index to reflect endocytosis efficiency. 4T1 cells internalized more iron from HFn/GA‐Fe compared to the GA‐Fe complex (Figure S8). We next performed a competitive assay to evaluate uptake of Cy5‐HFn by 4T1 in the presence of HFn/GA‐Fe. The Cy5 fluorescence intensity decreased with increasing HFn/GA‐Fe concentration (Figure 2C,D). The result detected by flow cytometry also further proved the receptor competition (Figure 2E). Conversely, free HFn was also employed to complete the uptake of HFn/GA‐Fe. The inhibition rate almost reached 50% when 400 µg/mL of free HFn was added (Figure 2F). We confirmed that the hybrid HFn/GA‐Fe still retained the targeting ability of HFn both in vitro and in vivo.

### 2.3 HFn/GA‐Fe Induced Tumor‐Specific Darkening and Hemorrhage

**FIGURE 3:**
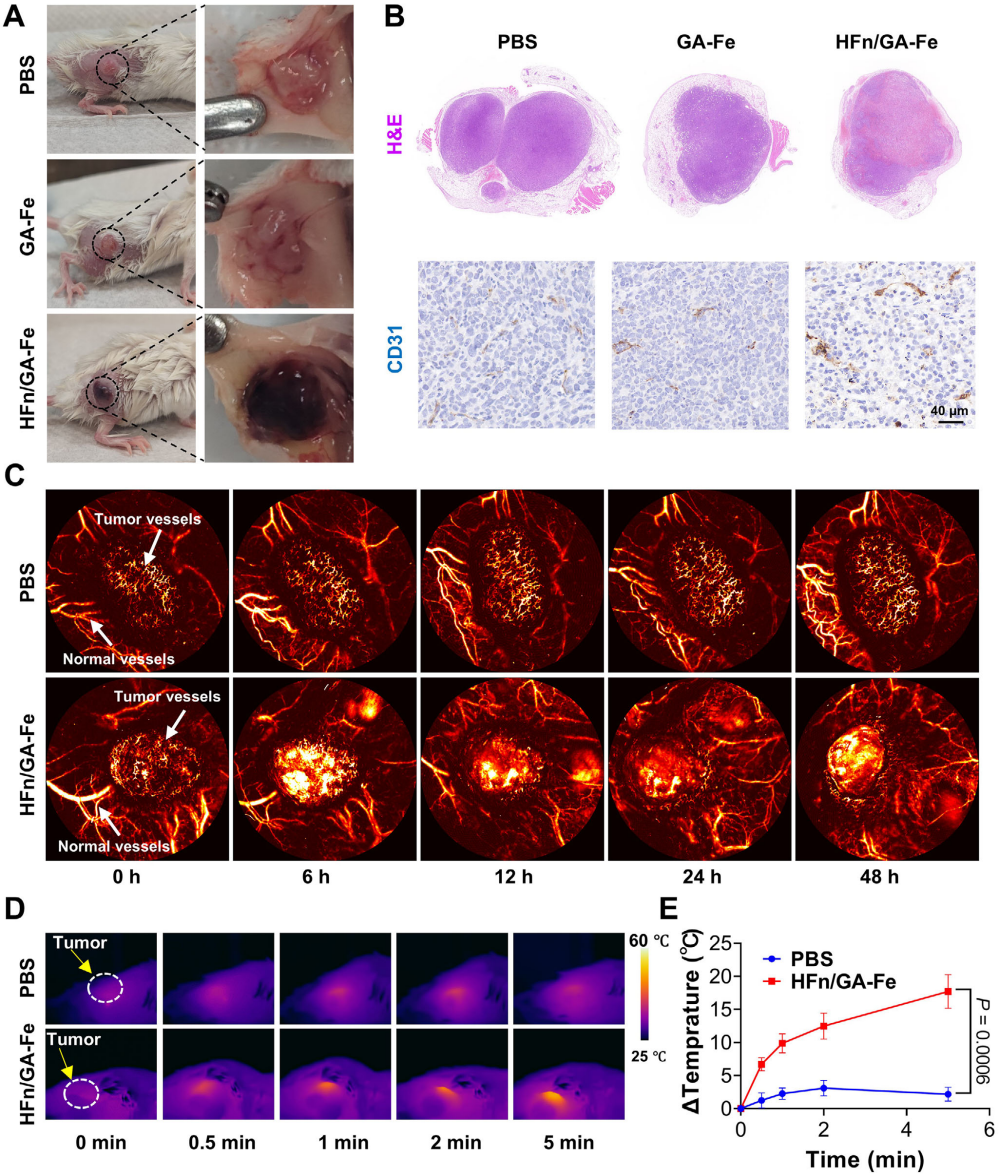
Tumor‐specific hemorrhage for enhanced PTT. (A) Images of 4T1 tumor at 12 h after intravenous injection of PBS, GA‐Fe, and HFn/GA‐Fe. (B) Whole H&E staining and CD31 staining of 4T1 tumor slices at 12 h after intravenous injection of PBS, GA‐Fe, and HFn/GA‐Fe. Scale bars: 40 µm. (C) Blood vessel images of tumors at 12 h post intravenous injection of PBS and HFn/GA‐Fe (n = 3). (D) Thermal imaging of 4T1 tumor at 12 h after intravenous injection of PBS, and HFn/GA‐Fe at 0, 0.5, 1, 2, 5 min under 660 nm laser irradiation with power of 0.75 W/cm^2^. (E) Temperature increasing curve at the tumor site during PTT (n = 3). Data are shown as mean ± SD. *P* stands for probability value used for hypothesis testing. All statistical analyses were performed using one‐way ANOVA.

After intravenous injection of PBS, GA‐Fe, and HFn/GA‐Fe, the tumors clearly turned a dark color in the HFn/GA‐Fe group at 12 h (Figure 3A). To identify the reason for this phenomenon, hematoxylin and eosin (H&E) staining and tumor vascular immunostaining (CD31) were performed on intact tumor tissues. Interestingly, the result showed that a large number of erythrocytes leaked into the tumor parenchyma and the structure of vascular endothelial cells was disrupted in the HFn/GA‐Fe group compared to other groups (Figure 3B). At 6 h post injection of HFn/GA‐Fe, the tumor blood vessels were significantly damaged as revealed by the photoacoustic imaging result (Figure 3C). To exclude the interference of tumor scabs, as scabs often form during tumor growth, tumors with or without scabs were compared after injection of PBS or HFn/GA‐Fe. The result showed the darkened color induced by HFn/GA‐Fe faded after 48 h, while the scab remained (Figure S9). No hemorrhage was found in normal organs, including the heart, liver, spleen, lungs, and kidneys as revealed by H&E staining (Figure S10). Considering the expression of TIM‐2 on brain endothelial, we also scanned the H&E staining of the mouse brain from the PBS group and HFn/GA‐Fe group. And no obvious morphological change was observed (Figure S11). In humans, 80% of the iron is stored in hemoglobin within erythrocytes [38]. Hence, we detected iron content by inductively coupled plasma mass spectrometry (ICP‐MS) to reflect erythrocyte numbers. The result showed iron content in tumors of the HFn/GA‐Fe group increased more than 4‐fold compared to PBS and GA‐Fe groups. The iron concentration in the spleen was also higher than that in other groups, which might be attributed to the clearance of damaged erythrocytes [39]. Iron concentration in other normal organs showed no significant differences, which further confirmed the tumor‐specific selectivity of HFn/GA‐Fe (Figure S12). LPS was reported to be capable of inducing tumor hemorrhage [40]. To rule out the possibility that residual LPS in the ferritin preparation induced hemorrhage, free ferritin was also injected, and no similar symptom was observed (Figure S13). Deoxyhemoglobin is a natural photothermal agent because of its absorption in the NIR region [41]. To assess the accumulation of hemoglobin, we conducted photoacoustic imaging at the tumor site (675 nm). The signal peaked at approximately 12 h post‐injection (Figure S14), which suggested the highest photothermal conversion efficiency. Therefore, 12 h post‐injection was set as the time point for tumor PTT. The tumor temperature during PTT was monitored by thermal imaging (Figure 3D). In the HFn/GA‐Fe group, the tumor temperature increased by more than 15°C after 5 min compared to ∼2°C in the PBS group under 660 nm irradiation at the power intensity of 0.75 W/cm^2^. Overall, HFn/GA‐Fe was able to induce tumor‐selective hemorrhage and enable tumor PTT (Figure 3E).

### 2.4 HFn/GA‐Fe Damaged Endothelial Cells Through ROS Production

**FIGURE 4:**
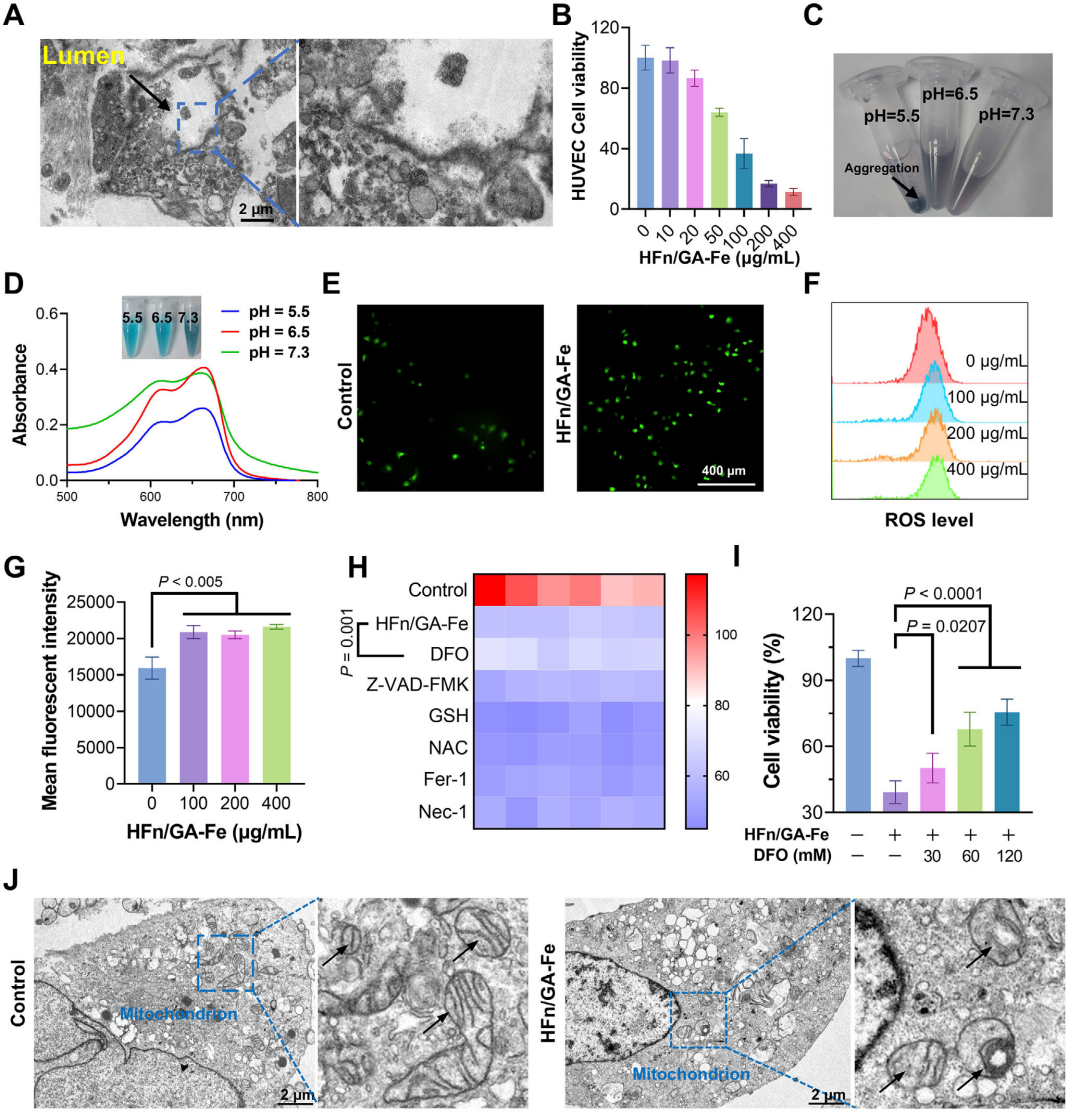
Damage to tumor vascular endothelial cells through the Fenton reaction. (A) TEM image of tumor slice at 12 h after HFn/GA‐Fe intravenous injection. Scale bars: 2 µm. (B) Cell viability of HUVECs at 24 h after incubation with 0, 10, 20, 50, 100, 200, 400 µg/mL of HFn/GA‐Fe (n = 5). (C) The dispersity of HFn/GA‐Fe under different pH values (5.5, 6.5, 7.3) at 24 h. (D) UV–vis absorption spectra of MB (10 µg/mL) at 2 h after incubation with HFn/GA‐Fe (200 µg/mL) and H_2_O_2_ (5 µmol) under pH5.5, 6.5, 7.3 at 37°C. (E) HUVEC cells were incubated with different concentrations of HFn/GA‐Fe for 6 h. Then the ROS was detected with a DCFH‐DA probe by fluorescent image and (F‐G) flow cytometry detection (n = 3). (H) Cell viability of HUVECs at 24 h after treatment with HFn/GA‐Fe (50 µg/mL) plus PBS, DFO (30 µm), Z‐VAD‐FMK (30 µm), GSH (2 mm), NAC (1 mm), Fer‐1 (2 µm), Nec‐1 (20 µm) (n = 6). (I) Cell viability of HUVECs with HFn/GA‐Fe (50 µg/mL) and DFO (0, 30, 60, 120 mm) (n = 6). (J) TEM images showed mitochondrial structural damage in HUVECs at 12 h after treatment with HFn/GA‐Fe (50 µg/mL). Scale bars: 2 µm. Data are shown as mean ± SD (n = 6). *P* stands for probability value used for hypothesis testing. All statistical analyses were performed using one‐way ANOVA.

We have demonstrated that the tumor darkening was due to the erythrocyte leakage, but the mechanism underlying tumor‐specific hemorrhage caused by HFn/GA‐Fe remained unclear. ROS have been reported to exert a direct effect on vascular endothelial cells [42], and GA‐Fe was able to generate ROS via the Fenton reaction [28]. The endothelial cell structure was disrupted as revealed by TEM (Figure 4A). We proposed that HFn/GA‐Fe might target tumor vascular endothelial cells and produce ROS therein. HFn/GA‐Fe induced over 50% cell death in human umbilical vein endothelial cells (HUVECs) at a concentration of 100 µg/mL (Figure 4B). However, the vascular‐disrupting function of HFn‐based drug delivery systems was not found in previous studies [22]. We speculated that these nanoparticles might be rapidly translocated to the basal side of endothelial cells and retained for a limited time [43]. Increasing particle size has been shown to prolong the retention time of nanoparticles in cells [44, 45]. Thus, HFn/GA‐Fe aggregation in endothelial cells might be the key mechanism. Considering the physicochemical changes that occur during transcytosis, we evaluated the dispersibility of HFn/GA‐Fe under different pH conditions (pH 5.5, 6.5, 7.3). HFn/GA‐Fe aggregated into precipitates under pH 5.5 (Figure 4C). After testing under a narrower pH gradient (0.1 pH unit increments), HFn/GA‐Fe was found aggregating at pH values below 5.5 (Figure S15). GA‐Fe did not aggregate at low pH, so we deduced this property was inherent to HFn. Hence, we examined the HFn status under different pH alone, and HFn also aggregated at pH 5.5 (Figure S16). Since proteins tend to aggregate at their isoelectric points (pIs), we predicted the pIs of ferritin heavy chain from human and other species (Table S1). All predicted pIs were approximately 5.5 (Figure S17). The sequence homology of ferritin heavy chain between *Chionodraco rastrospinosus*, *Xenopus laevis*, and *Homo sapiens* were only 71.66% and 68.18%, respectively (Table S1), which suggested the pI of ferritin heavy chain was highly conserved during evolution. This might explain the location of HFn in lysosomes, where the pH was around 5.5.

GA‐Fe has been shown to trigger the Fenton reaction, which catalyzes H_2_O_2_ into ·OH in previous studies [24]. The results of ultraviolet–visible spectroscopy (UV–vis) demonstrated that the highest Fenton activity occurred at pH 5.5 (Figure 4D), consistent with the findings from other studies [46]. Intracellular ROS production induced by HFn/GA‐Fe after incubation with HUVECs was also confirmed by fluorescent images (Figure 4E) and flow cytometry (Figure 4F,G). To further elucidate the mechanism by which ROS induces HUVECs death, various cell death inhibitors were used to reverse the cytotoxic effect. Only Deferoxamine mesylate (DFO) exerted a reversible protective effect (Figure 4H), and the reversibility can be enhanced with increasing DFO concentration (Figure 4I). DFO is a high‐affinity Fe (III) chelator clinically used to treat iron overload [47]. Thus, iron may be the key trigger of HUVECs' death. Additionally, the TEM observation revealed that the mitochondria in HUCEVs lost the normal structure compared to the normal group, including volume shrinkage and disorganized cristae (Figure 4J), which was consistent with the morphology change of mitochondria in ferroptosis [48]. In other studies, iron has also been found to damage blood vessels via excessive ROS production [49]. Collectively, our results revealed that iron played a key role in inducing ROS production and endothelial cell death.

### 2.5 PTT Induced Immunogenic Cell Death (ICD)

**FIGURE 5:**
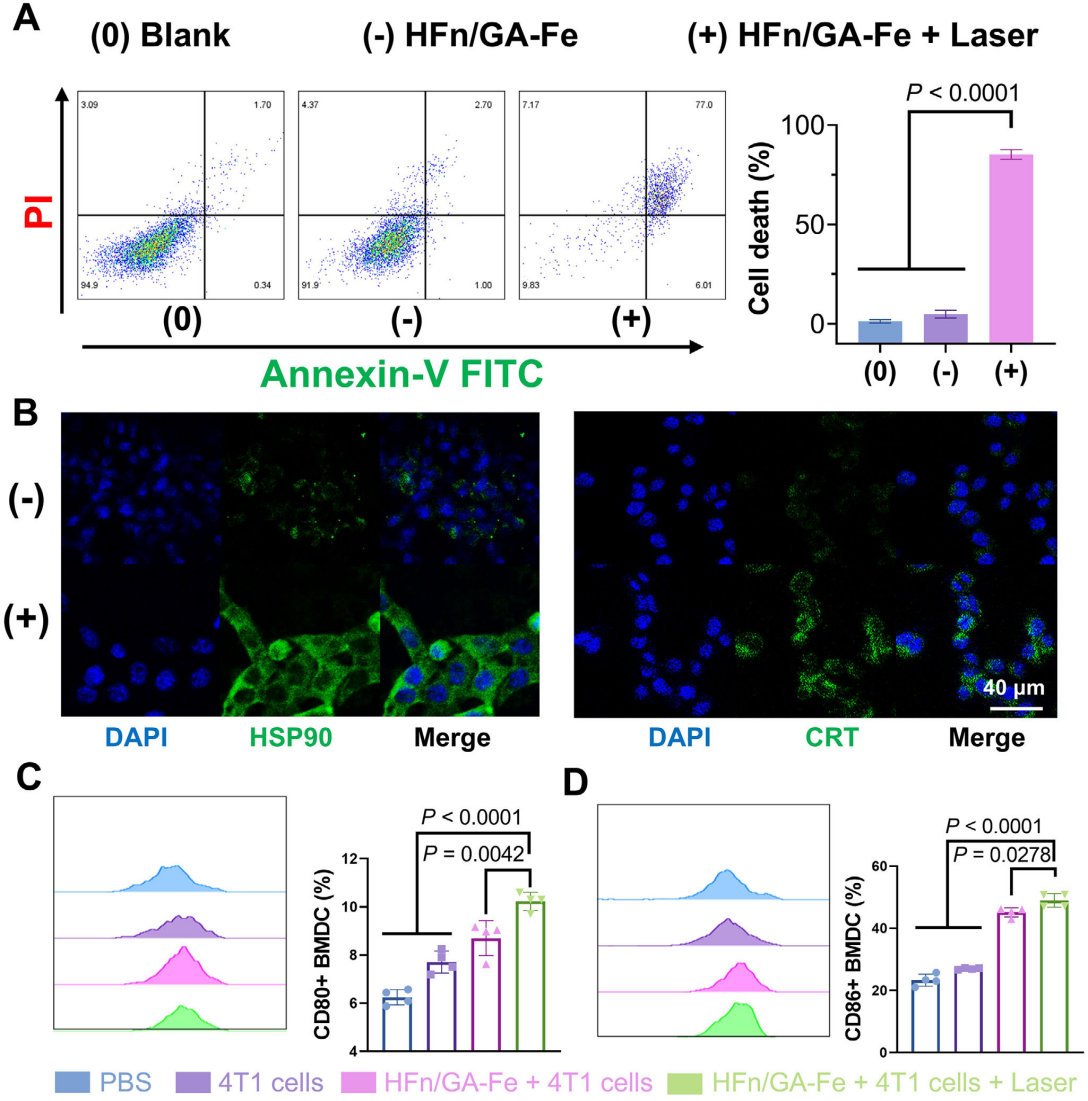
Immunogenetic cell death induced by PTT. (A) 4T1 cells treated with PBS, HFn/GA‐Fe (400 µg/mL), and HFn/GA‐Fe (400 µg/mL) + laser were stained with Annexin V‐FITC Apoptosis Detection Kit (Beyotime, Shanghai) and analyzed with cytometry at 6 h later (n ≥ 4). (B) HSP90 and CRT immunofluorescence of 4T1 cells treated with PBS, HFn/GA‐Fe (400 µg/mL), and HFn/GA‐Fe (400 µg/mL) + laser of before and post laser irradiation. Scale bars: 40 µm. (C,D) The CD80 and CD86 expression of BMDCs (1 × 10^5^ cells/well) at 24 h after incubated with 4T1 cells (500 µL) treated with 1640 medium (200 µL), HFn/GA‐Fe (200 µL, 1 mg/mL), and HFn/GA‐Fe + Laser (200 µL, 1 mg/mL) was detected by cytometry. Data are shown as mean ± SD (n = 5). *P* stands for probability value used for hypothesis testing. All statistical analyses were performed using one‐way ANOVA.

We evaluated the therapeutic efficacy of photothermal therapy (PTT) in 4T1 cells. Cell viability assays and live/dead staining demonstrated that nearly all 4T1 cells were killed, with nuclei stained by propidium iodide (PI), following laser irradiation at an HFn/GA‐Fe concentration of 400 µg/mL (Figure S18). Necrosis typically elicits robust immune responses and immune memory, which enhances anti‐tumor effects [50]. Annexin V‐FITC/PI staining showed a significant increase in cells undergoing late apoptosis and necrosis after photothermal treatment compared to the blank and laser‐free groups (Figure 5A). To further elucidate PTT‐induced immunogenic cell death (ICD) in 4T1 cells, several commonly used ICD markers were measured, including adenosine triphosphate (ATP), high Mobility Group Box 1 (HMGB1), calreticulin (CRT), and heat shock protein 90 (HSP90) [51]. Western blot (WB) results revealed upregulated expression of CRT and HSP90, alongside decreased HMGB1 levels (Figure S19)—a pattern consistent with the molecular signature of ICD [52]. Additionally, immunofluorescence (IF) assays were performed to analyze the localization and expression of these molecules within cells. The results indicated upregulated HSP90 expression, CRT exposure (Figure 5B), and HMGB1 release (Figure S20). As a damage‐associated molecular pattern (DAMP), extracellular ATP has been shown to induce dendritic cell (DC) maturation [53]. ATP detection assays confirmed that 4T1 cells released cytoplasmic ATP into the extracellular space following laser irradiation (Figure S21). DCs play a pivotal role in initiating anti‐tumor immunity, so DC activation is critical for effective anti‐tumor immune responses [54]. The expression of co‐stimulatory molecules (CD80 and CD86) increases during DC maturation. Thus, the expression of DC co‐stimulatory molecules was analyzed by flow cytometry (Figure S22). The results showed significantly higher CD80 (Figure 5C) and CD86 (Figure 5D) expression in DCs treated with irradiated 4T1 cells (pre‐incubated with HFn/GA‐Fe) compared to other groups. These findings confirm that PTT not only directly kills tumor cells but also triggers anti‐tumor immune responses. PTT‐induced ICD was also validated in animal models via WB and IF. WB results showed higher HSP90 and CRT expression, as well as lower HMGB1 levels, in the HFn/GA‐Fe + laser group (Figure S23). Furthermore, IF assays revealed stronger fluorescence intensities of HMGB1, HSP90, and CRT in this group compared to others (Figure S24). Collectively, PTT induces ICD in 4T1 cells and promotes the release of various DAMPs, thereby evoking anti‐tumor immunity.

### 2.6 Tumor Inhibition and Immunity Activation In Vivo

**FIGURE 6:**
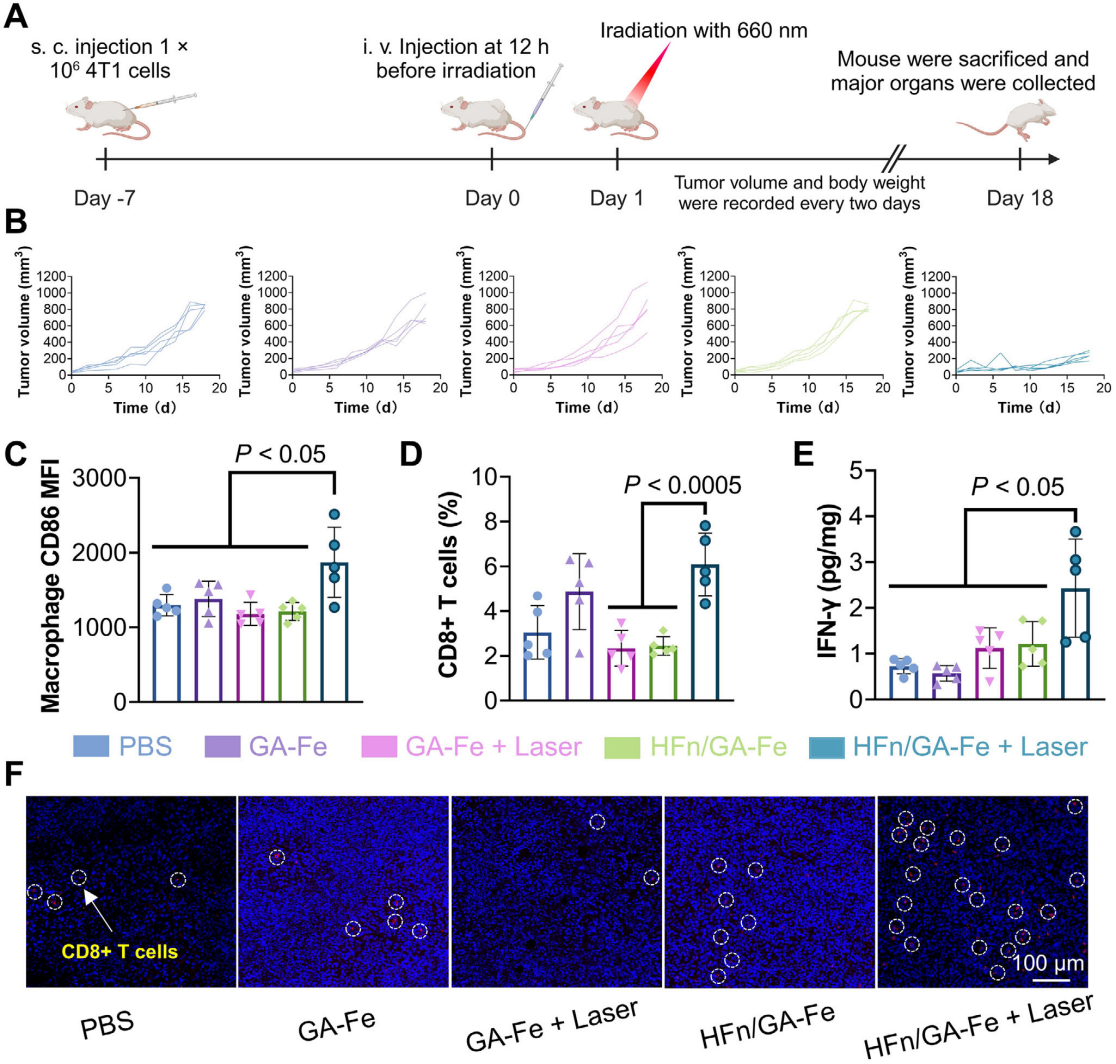
HFn/GA‐Fe induced photothermal ablation in 4T1 tumor model. (A) Schematic illustration of 4T1 tumor inoculation on day ‐7, drug intravenous injection on day 0, photothermal therapy on day 1, tumor volume and mouse weight recorded every two days, and mice sacrificed on day 18 (n = 5). (B) Individual tumor volume growth curve of each group with different treatment, including PBS, GA‐Fe, GA‐Fe + Laser, HFn/GA‐Fe, and HFn/GA‐Fe + Laser (n = 5). (C) The mean fluorescence intensity of macrophage CD86 in tumors with different treatment including PBS, GA‐Fe, GA‐Fe + Laser, HFn/GA‐Fe, and HFn/GA‐Fe + Laser (n = 5). (D) The proportion of CD45^+^ CD3^+^ CD8^+^ cells in the tumors at day 18 of different groups after treatment (n = 5). (E) IFN‐γ expression of tumor lysis supernatant after homogenization was detected by ELISA (n = 5). (F) CD8^+^ T cells staining in different groups. Scale bars: 100 µm. Data is shown as mean ± SD. *P* stands for probability value used for hypothesis testing. All statistical analyses were performed using one‐way ANOVA.

We evaluated the anti‐tumor efficacy of HFn/GA‐Fe using a 4T1 tumor model, with all mice receiving a single treatment (Figure 6A). The tumor volume in the HFn/GA‐Fe + laser group was only one‐fourth of that in the control group (Figure 6B, Figures S24A–C), and all mice exhibited minimal fluctuations in body weight (Figure S25D). Terminal deoxynucleotidyl transferase dUTP nick end labeling (TUNEL) staining revealed a higher number of apoptotic cancer cells in the HFn/GA‐Fe + laser group (Figure S26). No obvious damage was observed in other normal tissues (Figure S27), confirming the safety of this therapeutic strategy. Besides, we tested the short‐term toxicity at 12 h post one treatment and long‐term toxicity on the eighth day post four treatments with HFn/GA‐Fe. The parameters in both were stayed in reference range, dictating no obvious systematic toxicity was observed (Table S2). Anti‐tumor immunity also plays a crucial role in PTT, as it can further inhibit tumor growth after irradiation. Flow cytometry was used to analyze CD86^+^ M1‐like macrophages and CD8^+^ cytotoxic T cells within tumors (Figures S28 and S29). Following HFn/GA‐Fe + laser treatment, the proportions of CD8^+^ T cells and M1‐like macrophages in tumors were significantly higher than those in the other groups (Figure 6C,D). Interferon‐gamma (IFN‐γ) can induce cancer cell apoptosis and modulate immune responses [55, 56]. Enzyme‐linked immunosorbent assay (ELISA) showed that the tumor IFN‐γ concentration was the highest in the HFn/GA‐Fe + laser group (Figure 6E). Furthermore, CD8 immunofluorescence staining of tumor sections demonstrated increased infiltration of CD8^+^ T cells into tumors after PTT (Figure 6F), consistent with the findings from similar studies [51]. Collectively, PTT enhanced by tumor hemorrhage effectively elicits anti‐tumor immune responses in vivo.

### 2.7 Tumor Inhibition by Chemo‐PTT

**FIGURE 7:**
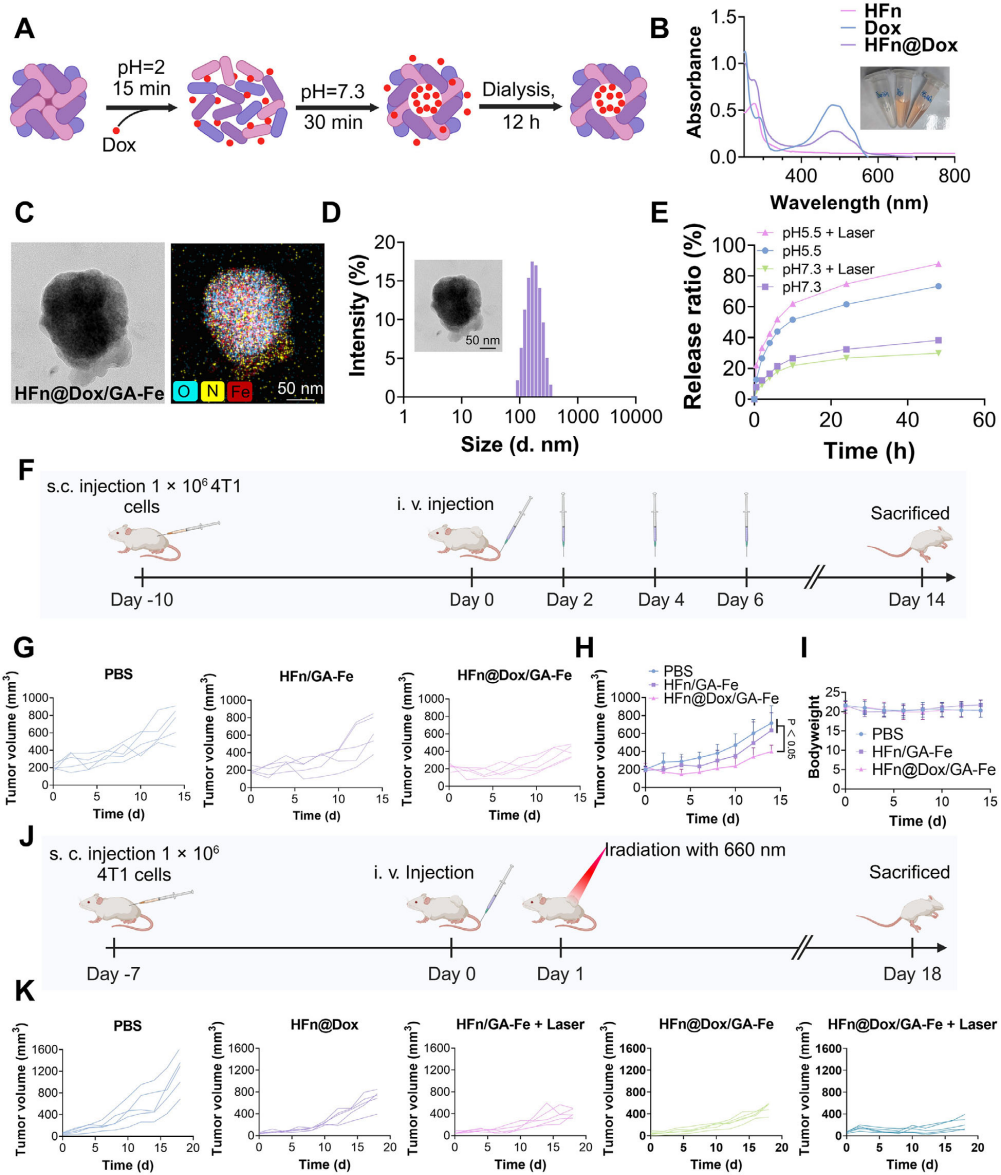
Chemotherapy drug encapsulated in ferritin for combination therapy. (A) Schematic diagram of Dox loading into Ferritin by pH‐adjustive drug loading method. (B) UV‐Vis absorption spectra of Dox, HFn, and HFn@Dox. (C) TEM image and O, N, Fe element mapping of HFn@Dox/GA‐Fe. (D) Size distribution of HFn@Dox/GA‐Fe measured by DLS. (E) Dox release curve including 0, 0.5, 2, 4, 6, 10, 24, 48 h of HFn/GA‐Fe under pH 5.5, pH 5.5 + Laser, pH 7.3, pH 7.3 + Laser. (F) Schematic diagram of vascular destruction combined with chemotherapy, including 4T1 tumor inoculation on day ‐10, intravenous injection of PBS, HFn@Dox/GA‐Fe on day 0, 2, 4, 6, and mice sacrifice on day 14. (G) Individual tumor volume growth curve of 4T1 mice tumor after being treated with PBS, HFn/GA‐Fe. HFn@Dox/GA‐Fe (n = 5). (H) Tumor volume growth curve after being treated with PBS, HFn/GA‐Fe NPs, and HFn@Dox/GA‐Fe (n = 5). (I) Mean mouse body weight curve after being treated with PBS, HFn/GA‐Fe NPs, and HFn@Dox/GA‐Fe (n = 5). (J) Schematic diagram of chemo‐PTT including 4T1 tumor inoculation on day ‐1, intravenous injection of drug on day 0, laser irradiation on day 1, and mice sacrifice on day 18 (n = 5). (K) Individual tumor volume growth curve of 4T1 tumor after being treated with PBS, HFn@Dox, HFn/GA‐Fe + Laser, HFn@Dox/GA‐Fe, and HFn@Dox/GA‐Fe + Laser (n = 5). Data are shown as mean ± SD. *P* stands for probability value used for hypothesis testing. All statistical analyses were performed using one‐way ANOVA.

Chemotherapy is widely combined with photothermal therapy (PTT) to prevent cancer recurrence [57]. Given the cavity of ferritin nanocages, HFn/GA‐Fe can encapsulate chemotherapeutic drugs to enhance therapeutic efficacy. Doxorubicin (Dox), a first‐line chemotherapeutic agent for breast cancer [58], was loaded into HFn via pH adjustment (Figure 7A). Ultraviolet–visible (UV‐Vis) absorption spectra confirmed the successful loading of Dox into HFn (Figure 7B). Based on calculations from the fluorescence fitting curve, each HFn molecule loaded approximately 23 Dox molecules (Figure S30). HFn@Dox was used instead of HFn to react with GA‐Fe, forming a nanoparticle designated HFn@Dox/GA‐Fe. The morphology of HFn@Dox/GA‐Fe was observed by transmission electron microscopy (TEM), and O, Fe, and N were detected in HFn@Dox/GA‐Fe (Figure 7C). Its hydrodynamic diameter was measured to be ∼184.1 nm (PDI = 0.297) (Figure 7D). HFn@Dox/GA‐Fe exhibited accelerated Dox release under conditions of pH 5.5 and laser irradiation (Figure 7E). Prior to combining with PTT, we evaluated the vascular‐disrupting and chemotherapeutic effects of HFn/GA‐Fe and HFn@Dox/GA‐Fe in a 200 mm^3^ 4T1 tumor model with four treatments (Figure 7F). HFn@Dox/GA‐Fe effectively inhibited tumor growth (Figure 7G,H), highlighting the advantages of chemotherapy in systemic therapy. HFn/GA‐Fe also exerted anti‐tumor effects mediated by tumor vascular destruction (Figure 7G,H; Figure S31). No significant weight loss was observed during treatment (Figure 7I). Subsequently, chemotherapy was combined with PTT (Figure 7J), and HFn@Dox/GA‐Fe + laser significantly inhibited tumor growth with a single treatment (Figure 7K; Figure S32), demonstrating the synergistic effect of chemo‐PTT. Furthermore, the HFn@Dox/GA‐Fe group exhibited superior anti‐tumor efficacy compared to the HFn@Dox group, which may be attributed to its vascular‐disrupting effect.

## 3 Conclusion

In our work, we developed a novel vascular‐disrupting agent through a simple reaction between GA‐Fe and ferritin. HFn could bind with TfR1/TIM‐2 to mediate precise tumor targeting and rapid particle internalization into endothelial cells. The darkening of tumor color in mouse models could be attributed to the aggregation and ROS production of HFn/GA‐Fe in lysosomes of tumor vascular endothelial cells. HFn/GA‐Fe induced tumor‐specific hemorrhage and enhanced in situ PTT to evoke anti‐tumor immunity. By combination with chemotherapy, the therapeutic effect further improved. Notably, HFn/GA‐Fe exhibited superior tumor selectivity with little retention and non‐toxicity in normal organs. Such a ferritin‐based therapeutic approach may easily be translated into clinical trials.

## 4 Experimental Section

### 4.1 Total RNA Extraction and cDNA Synthesis

Total RNA was extracted from HepG2 using an RNAeasy Animal RNA Isolation Kit (Beyotime, Shanghai). After that, the cDNA first strand was synthesized using PrimeScript II first Strand cDNA Synthesis Kit (Takara, Beijing) with mRNA (2 µg) as templates. The production was stored at ‐80°C for the following experiments.

### 4.2 Plasmid Construction and Gene Recombinant Expression

ORF of the Human ferritin heavy chain gene was amplified with expression primers (FRf: TACTCAGAATTCATGACGACCGCGTCCACC; FRr: TACTCACTCGAGTTAGCTTTCATTATCACT). Then the ferritin gene fragment and PET30a plasmid were both digested with EcoRI and XhoI at 37°C for 3 h and linked with T4 ligase at 16°C overnight. The ligation product was transformed into compete Rosetta cell. The positive strain, which was proved by sequencing, was inoculated into LB liquid medium overnight and transferred into fresh LB medium at a volume ratio of 1:100. When the bacterial growth reached the logarithmic phase, Isopropyl‐β‐d‐thiogalactoside (IPTG) (0.5 mmol) was added. The bacteria were collected and ultrasonicated after 4 h. The supernatant was heated at 70°C for 10 min. And then the remaining supernatant was salted out with 60% Ammonium sulfate (NH_4_SO_4_) (19.50 g in 50 mL). The sediment was resuspended with PBS and dialyzed in PBS overnight. And the endotoxin was removed using the phase separation method [59] before use, and the concentration was measured with the Enhanced BCA Protein Assay Kit (Beyotime, Shanghai).

### 4.3 The Fluorescent Labeling of Ferritin

In brief, Cy5‐NHS was added into HFn solution (0.1 м NaHCO_3_, pH 8.5) at the molar ratio of 10: 1. The solution was stirred overnight at 4°C, avoiding light. The free dye was removed by dialysis with a 3000 Da dialysis bag. The concentration of labeled Cy5 was determined by measuring the absorbance at 673 nm, and the concentration of HFn was determined with the Enhanced BCA Protein Assay Kit (Beyotime, Shanghai). The Cy5‐labeled protein was stored at ‐20°C for subsequent use.

### 4.4 Synthesis of HFn/GA‐Fe

The nanoparticles were synthesized by a two‐step reaction. Firstly, FeCl_2_·4H_2_O (30 mg) and gallic acid (GA) (20 mg) were dissolved in H_2_O (10 mL) and stirred overnight at room temperature. Then the GA‐Fe solution was mixed with 5 mL 1 mg/mL HFn solution and stirred for 0.5 h. The product was washed with H_2_O three times by centrifugation and resolved in PBS under ultrasonication. To accurately measure the concentration of HFn/GA‐Fe by absorption value, the fitting curve between OD600 and concentration was calculated by Prism 8.0.

### 4.5 Characterizations of HFn/GA‐Fe

The hydrodynamic diameter, polydispersity index (PDI), and homodisperse potential of HFn/GA‐Fe were measured by dynamic light scattering (DLS, Malvern Zetasizer Nano ZS, UK) at 25°C. The morphology was observed by transmission electron microscope (TEM, FEI Talos F200X, USA). The absorption spectrum of HFn/GA‐Fe was measured by Ultraviolet‐visible Spectrophotometer (UV–vis Spectrophotometer, Hach DR6000, German). The photoacoustic imaging signal was detected by Photoacoustic tomography. Thermal imaging was pictured by the FOTRIC thermal imager.

### 4.6 Investigation of the ROS Generation Ability of Nanoparticles

To detect the Fenton reaction between HFn/GA‐Fe and H_2_O_2_ under different pH. The absorbance of Methylene Blue (MB) was set as the indicator. In brief, HFn/GA‐Fe (200 µg/mL) were incubated with H_2_O_2_ (5 µmol) and MB (10 µg/mL) under pH 5.5, 6.5, 7.3 at 37°C for 2 h. The absorbance scan was analyzed with UV spectrophotometer.

### 4.7 Cell Uptake of HFn/GA‐Fe by ICP‐MASS

4T1 cells (CL‐0007, Procell system) were seeded into a 24‐well plate at a density of 5 × 10^4^ cells/well. The next morning, HFn/GA‐Fe and GA‐Fe were added to the plate till the final concentration of iron content reached 10 µg/mL and incubated for 0, 0.5, 1, 2, and 4 h. Then the cells were washed with PBS three times and collected. After re‐suspended with 400 µL aqua regia, the solution will be placed in a fume hood overnight with the tube opening. The next morning, the reaction liquid was supplemented with 2% aqueous nitric acid (10 mL) and filtered by 0.22 µm Millipore before being detected by iCAP Q ICP‐MASS (Thermal, America).

### 4.8 The Cell Uptake Inhibition of HFn/GA‐Fe and HFn

The cell seeding was the same as in the above experiment. Free HFn was added at different final concentrations (0, 50, 100, 200, and 500 µg/mL) at 0.5 h before the supplement of 100 µg/mL HFn/GA‐Fe. After 1 h, the cells were treated referred to the last experiment and detected by iCAP Q ICP‐MASS (Thermal, America). To determine whether the HFn/GA‐Fe were internalized by 4T1 cells through the same receptor of HFn. HFn/GA‐Fe were also utilized to compete with Cy5‐HFn for cell uptake. In brief, Cy5‐HFn (10 µg/mL) and HFn/GA‐Fe of different concentrations of 20, 50, 100, and 200 µg/mL were co‐incubated with 4T1 cells for 0.5 h and terminated with pre‐cooled PBS. Then, the cells were by fluorescent microscopy and detected in flow cytometry (CytoFLEX LX Flow Cytometers, America).

### 4.9 Isoelectric Point (pI) Prediction

To examine whether the pI of HFn was about pH 5.5, pI of the heavy ferritin chain from various species was predicted. First, the protein sequences of Homo sapiens, Rattus norvegicus, Mus musculus, Bos taurus, Felis catus, Anser cygnoides, Chionodraco rastrospinosus, Xenopus laevis, and Pongo abelii were downloaded from NCBI (https://www.ncbi.nlm.nih.gov/). And then the pI was predicted in Expasy (https://web.expasy.org/protparam/).

### 4.10 DCFH‐DA Probe Assay

ROS production is one of the characteristics of ferroptosis, and to better mimic the blood vessels, we used the HUVEC cells as the model cells. In brief, HUVEC cells were incubated with different concentrations of HFn/GA‐Fe for 6 h. Then the ROS was detected with a DCFH‐DA probe (Beyotime, Shanghai).

### 4.11 Cell Death Inhibition Assay

To find out the key factor by which HFn/GA‐Fe induced cell death, cell death inhibitors, including PBS, DFO (30 µm), Z‐VAD‐FMK (30 µm), GSH (2 mM), NAC (1 mm), Fer‐1 (2 µm), and Nec‐1 (20 µm) were preincubated with HUVEC cells for 1 h before HFn/GA‐Fe (50 µg/mL) was added. The cell viability was detected with Cell Counting Kit‐8 (CCK‐8) after 24 h.

### 4.12 Cell Viability Analysis

4T1 cells were seeded in a 96‐well plate at a density of 1 × 10^4^ cells per well. The next day, different concentrations of HFn/GA‐Fe were added to different wells, and part of the wells were treated with a 660 nm laser at a power of 1.5 W/cm^2^ for 5 min. After 6 h, the cell viability was detected by CCK‐8, and apoptosis was analyzed with Annexin V‐FITC Apoptosis Detection Kit (Beyotime, Shanghai).

### 4.13 Calcein/PI Cell Staining and Annexin V‐FITC/PI Cell Staining

4T1 cells were irradiated with a 660 nm Laser after incubated with HFn/GA‐Fe (400 µg/mL). Then the cells were stained with Calcein/PI Cell Viability/Cytotoxicity Assay Kit (Beyotime, Shanghai) and Annexin V‐FITC Apoptosis Detection Kit (Beyotime, Shanghai) 6 h later. And the result was observed and photographed by DMI8 fluorescence microscope (Leica, Wetzlar, Germany).

### 4.14 Immunofluorescence (IF) Assay of DAMPs in 4T1 Cells

First, 5 × 10^4^ cells were plated in each well of a 24‐well plate and adhered to the cell slide overnight. The next day, the slides were divided into HFn/GA‐Fe and HFn/GA‐Fe + laser groups. After the same photothermal treatment as above, the cell slides were fixed with 4% paraformaldehyde and washed with PBST three times. After that, 10% of normal goat serum was dropped to block for 1 h at room temperature. Then the diluted DAMP antibodies (1:500 in PBST), including HMGB1 antibody (Beyotime, AF5192) (12 h post‐irradiation), HSP90 antibody (Proteintech, 13171‐1‐AP) (12 h post irradiation), and CRT antibody (Beyotime, AF1666) (4 h post irradiation) were dropped on the cell slides and incubated at 4°C overnight. After washing again, AF488‐labeled Goat Anti‐Rabbit IgG (H+L) (1:1000 in PBST) (Beyotime, A0423) was dropped and kept at room temperature for 1 h. Then, 1 µg/mL DAPI solution was added dropwise and washed 10 min later. Finally, the images were collected with a confocal laser scanning microscope (CLSM) (Leica, Wetzlar, Germany).

### 4.15 Western Blot of DAMPs in 4T1 Cells

First, 5×10^4^ cells were plated in each well of a 24‐well plate and adhered overnight. The next day, the wells were divided into three groups, including PBS, HFn/GA‐Fe, and HFn/GA‐Fe + laser groups, including three repeats of each group. After the nanoparticles were added, the HFn/GA‐Fe + laser groups were treated with 1.5 w/cm^2^ 660 nm laser for 5 min. 12 h later, the total protein of each group was extracted with RIPA solution containing PMSF (10 mm) by lysis for 30 min and quantified by BCA assay. Then the expression of DAMPs in each group was detected by western blot (WB) with different antibodies, including HMGB antibody, CRT antibody, HSP90 antibody, and GAPDH antibody as the internal reference.

### 4.16 PTT Induced BMDC Maturation

4T1 cells were collected and resuspended to 5 × 10^5^ cells/mL. Then the 4T1 cells were divided into three groups, including 500 µL cells solution treated with 1640 medium (200 µL), HFn/GA‐Fe (200 µL, 1 mg/mL), HFn/GA‐Fe + Laser (200 µL, 1 mg/mL), respectively. After treatment, each group (100 µL) was added to a U‐shaped 96‐well plate containing BMDC (1 × 10^5^ cells/well) and kept for 24 h. Subsequently, BMDC was piped out and washed with PBS before being blocked with PBS containing 1% BSA for 15 min. Then each sample was washed again with PBS and incubated away from light with APC‐CD11c antibody (0.25 µg/sample) (Biolegend, N418), FITC‐CD80 antibody (0.25 µg/sample) (Biolegend, 16‐10A1), and PE‐CD86 antibody (0.25 µg/sample) (Biolegend, GL‐1) for 30 min at 4°C. Finally, the cells were resuspended with 200 mL of PBS and detected by flow cytometry after washing away the excess antibodies.

### 4.17 Visualization of HFn Targeted Tumors in Zebrafish

The zebrafish tumor model was built according to the previously reported microinjection protocol in our group [60]. In brief, the 4T1 cells were stained with DiO (10 µg/mL) solution. And approximately DiO labeled 4T1 cells (1 × 10^6^ cells/50 µL, 5 nL) were injected into the fish larvae 48 h post fertilization (hpf) beneath the yolk sac. Embryos were screened for successful transplantation and maintained in a 28°C incubator for 24 h. Subsequently, Cy5 HFn NPs (∼ 1 mg/ml HFn, 0.36 mg/ml Cy5, 5 nL) and free Cy5‐NHS were intravenously injected into the posterior cardinal vein near the swimming bladder of zebrafish larvae with a Nanoject III (Drummond Scientific Company, USA) and a stereomicroscope (Olympus, Japan). Finally, the zebrafish were imaged using a DMI8 fluorescence microscope (Leica, Wetzlar, Germany). Fluorescence imaging settings for DiO detection were Ex: 475 nm/Em: 501 nm, and those for Cy5‐NHS detection were Ex: 635 nm/ Em: 670 nm.

### 4.18 Building of the 4T1 Tumor Model and Photoacoustic Imaging, Vessels Image of Tumors

Balb/C mice were cultured in a Specific Pathogen‐Free (SPF) animal facility at a density of 4–5 mice per cage with enough standard food and water. After 4T1 cells were cultured to log phase, the cells were treated as above and resuspended to a final concentration of 1 × 10^7^ cells/mL in PBS. Then 4–6 weeks female Balb/C mice were inoculated subcutaneously on the right flank with a cell suspension (100 µL). HFn/GA‐Fe (200 µL) at a dosage of 10 mg/kg and PBS (200 µL) were injected intravenously when the tumor volume reached 50 mm^3^. After anaesthetizing with isoflurane, the photoacoustic signal of tumors in different mice was recorded by a home‐made Photoacoustic tomography device and optical resolution photoacoustic microscopy (OR‐PAM) system (VIS‐50, PAOMTek Inc., Chengdu) at time points of 0, 6, 12, 24, 36, and 48 h post‐injection. The intensities were calculated by software image J and plotted by software Prism 8.0.

### 4.19 Iron Content Detection by ICP‐MASS

4T1 tumor‐bearing mice were injected intravenously with PBS (200 µL), GA‐Fe (iron content: 84.5 µg/mL, 200 µL), and HFn/GA‐Fe (iron content: 80.3 µg/mL, 200 µL), respectively. The mice were sacrificed 12 h later via CO_2_ chamber euthanasia. Organs, including the heart, liver, spleen, lung, kidney, and tumors of each mouse, were collected by surgery. The tissues were digested in 70% Nitric acid with the microwave. The digestion product was adjusted to a constant volume of 50 mL before the iron detection by the ICP‐MASS machine.

### 4.20 Western Blot of DAMPs in 4T1 Tumor

The mice were divided into 6 groups: PBS (200 µL), PBS + Laser (200 µL), GA‐Fe (200 µL), GA‐Fe + Laser (200 µL), HFn/GA‐Fe (1 mg/mL, 200 µL), and HFn/GA‐Fe + Laser (1 mg/mL, 200 µL). 12 h after treatment, the mice were sacrificed via CO_2_ chamber euthanasia and tumor tissues were collected. Then the total protein of tumors was extracted by tumor homogenization in radioimmunoprecipitation assay buffer and centrifugation. And the protein concentration was quantified with the BCA kit (Beyotime, Shanghai). Then the expression level of different immunogenetic cell death markers, including HSP90, HMGB1, and CRT were analyzed by western blot with β‐actin as the internal reference.

### 4.21 Immunofluorescence Assay of DAMPs in 4T1 Tumor

The tumors from the above six different groups were first frozen and sectioned. The slides were fixed with 4% paraformaldehyde for 15 min at room temperature. 10% of normal goat serum was dropped on the slides and incubated for 1 h at after washing the slides with PBS. Subsequently, the diluted DAMP antibody mentioned previously was dropped on the cell slides and followed the same method as in the cell‐level assay.

### 4.22 Tumor PTT

4–6 weeks‐old female Balb/C mice were divided into five groups (n = 5): PBS (200 µL), GA‐Fe (200 µL), GA‐Fe + laser (200 µL), HFn/GA‐Fe (1 mg/mL, 200 µL), and HFn/GA‐Fe + Laser (1 mg/mL, 200 µL). The tumor model was established in the same way mentioned previously. The mice were intravenously injected with HFn/GA‐Fe at 10 mg/kg and other drugs with the same iron concentration when the tumor volume reached 50 mm^3^. 12 h later, the mouse tumors of the laser groups were irradiated under 660 nm laser at 0.75 W/cm^2^ for 5 min under gas anesthesia with isoflurane. After therapy, tumor volumes were measured by vernier calipers according to the formula “V = 1/2×a×b^2, V: volume, A: length, B: width”. Mouse weights were measured by electronic scales every other day. When the largest tumor size of the PBS group reached around 1500 mm^3^, all mice were sacrificed via CO_2_ chamber euthanasias and both tumors and other organs were stored at −80°C.

### 4.23 Immunity Analysis After PTT

The tumors of each group were cut into pieces and digested with PBS containing 10% FBS, 1 mg/mL collagenase, 0.1 mg/mL DNase I, and 0.1 mg/mL hyaluronidase at 37°C for 1 h. After that, the tissue blocks were ground and filtered with a 70 µm cell strainer. Then CD3‐FITC (0.25 µg/sample), CD8‐APC (0.25 µg/sample), and CD11b‐FITC (0.25 µg/sample), CD80‐APC (0.25 µg/sample) were employed to stain T‐cytotoxicity cells and M1‐like macrophages following the method mentioned above. Then the cell suspensions were analyzed by a BD LSRFortessa flow cytometer (Becton, Dickinson Company, NJ, USA) after washing.

### 4.24 ELISA Assay for Cytokines Analysis

The tumor tissue was cut and weighed. Then the tissue was homogenized in RIPA with 1 mm PMSF. Then the concentration of IFN‐γ was measured with an ELISA kit (Biolegend, USA).

### 4.25 Hematoxylin‐Eosin (H&E) Staining of Organs

Organs, including the heart, liver, spleen, lung, and kidney, were fixed in 4% paraformaldehyde for more than 24 h. And subsequent histological section and H&E staining were finished by Wuhan Servicebio Technology Co., Ltd (Wuhan, China).

### 4.26 Dox Encapsulation in HFn

The method of loading Dox in HFn referred to the previously reported PH assembly/disassembly mechanism [61]. In brief, the HFn (1 mg/mL) was dissolved in NaCl solution (150 mm), and then the pH was adjusted to 2. Dox was added (0.2 mg/mL). After 15 min, the pH was adjusted to 7 and stirred for another 2 h. The free Dox was excluded by dialysis in PBS (2 L). Finally, protein concentration was determined with a BCA kit, and the encapsulated drug was measured by an ultraviolet spectrophotometer compared to a predetermined Dox calibration curve.

### 4.27 Tumor Growth Inhibition by HFn@Dox/GA‐Fe

The 4T1 tumor model was established the same as mentioned previously. When the tumor volume reached 200 mm^3^, the mice were divided into three groups: PBS, HFn/GA‐Fe, and HFn@Dox/GA‐Fe group. The different groups were injected with PBS (200 µL), HFn/GA‐Fe (1 mg/mL, 200 µL) and HFn@Dox/GA‐Fe (1 mg/mL, 200 µL) at days 0, 2, 4, and 6 respectively. The tumor volume and body weight of each mouse were recorded every two days. When the tumor volume in PBS reached around 1500 mm^3^, the experiment was stopped, and tumors were collected and weighed.

### 4.28 Tumor Chemo‐PTT Combination Therapy

The experiment process was similar to the PTT, and the groups were changed into: PBS, HFn@Dox, HFn@Dox/GA‐Fe, HFn/GA‐Fe +Laser, and HFn@Dox/GA‐Fe + laser groups. The tumor volume, tumor weight, and body weight were recorded as mentioned above.

### 4.29 Ethical Issues

All zebrafish and mice experiments were conducted following the protocols (UMARE‐038‐2023, wide type zebrafish), (UMARE‐014‐2022: BALB/c mice) approved by the Institutional Animal Care and Use Committee of the University of Macau, respectively. Female BALB/c mice (4–6 weeks old) were purchased from the Faculty of Health Sciences of the University of Macau. The maximum tumor volume of mice in the tumor‐related animal experiments in this study did not exceed the allowable range. Zebrafish were bred in accordance with the standard operational guidelines (http://zfin.org).

### 4.30 Statistical Analysis

All experiment data were displayed as the mean ± SD and analyzed with GraphPad Prism 8 software. Unpaired, two‐tailed t‐tests were employed to compare the difference between two independent groups, and one‐way ANOVA was employed to compare the difference among three or more independent groups. *P* < 0.05 was considered to be statistically significant.

## Conflicts of Interest

The authors declare no conflict of interest.

## Supporting information




**Supporting File**: adhm70830‐sup‐0001‐SuppMat.docx.

## Data Availability

The data that support the findings of this study are available from the corresponding author upon reasonable request.
