# Supplementary material for: Ferritin‐Doped Nanoparticles Triggered Tumor‐Specific Darkening for Enhanced Photothermal Tumor Ablation and Immune Activation
Source: Adv Healthc Mater. 2026 Jan 24;15(14):e05119. doi: 10.1002/adhm.202505119 (PMC13068299; doi:10.1002/adhm.202505119)
Supplement: Supplementary file 1 — Supporting File: adhm70830‐sup‐0001‐SuppMat.docx. [file ADHM-15-0-s001.docx]

**Ferritin-doped nanoparticles triggered tumor-specific darkening for enhanced photothermal tumor ablation and immune activation**

*Haidong Zha, Xiao Liang, Long Xi, Jiamao Chen, Wenzhao Zhong, Zhen Yuan, Meng Xu,* Ying Zheng**

H. Zha, J. Chen, W. Zhong, M. Xu, Y. Zheng

State Key Laboratory of Mechanism and Quality of Chinese Medicine

Institute of Chinese Medical Sciences

University of Macau

Macau 999078, China

E-mail: mengxu@um.edu.mo (M. Xu), yzheng@um.edu.mo (Y. Zheng)

Y. Zheng

Guangdong-Hong Kong-Macao Joint Laboratory for New Drug Screening

University of Macau

Taipa, Macau 999078, China

Y. Zheng, X. Liang, Z. Yuan,

Faculty of Health Sciences

University of Macau

Macau 999078, China

L. Xi

School of Pharmaceutical Sciences

Guangzhou University of Chinese Medicine

Guangzhou 510006, China

Keywords: human ferritin heavy chain, Transferrin receptor 1, Fenton reaction, vascular disrupting agent, photothermal therapy


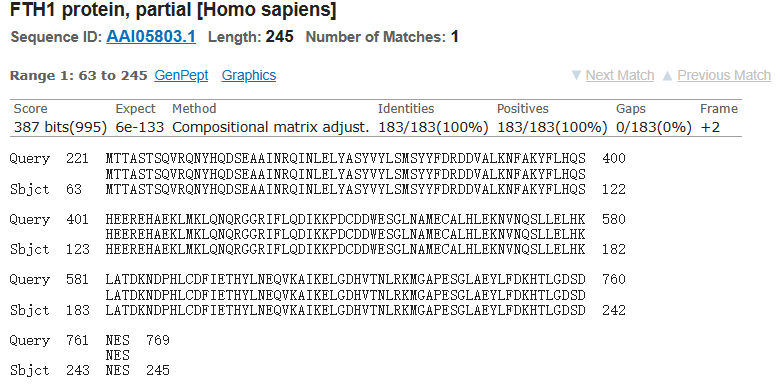


**Figure** **S1**. Alignment between sequencing result and FTH1 protein sequence of human by blastx in NCBI (https://blast.ncbi.nlm.nih.gov/Blast.cgi).


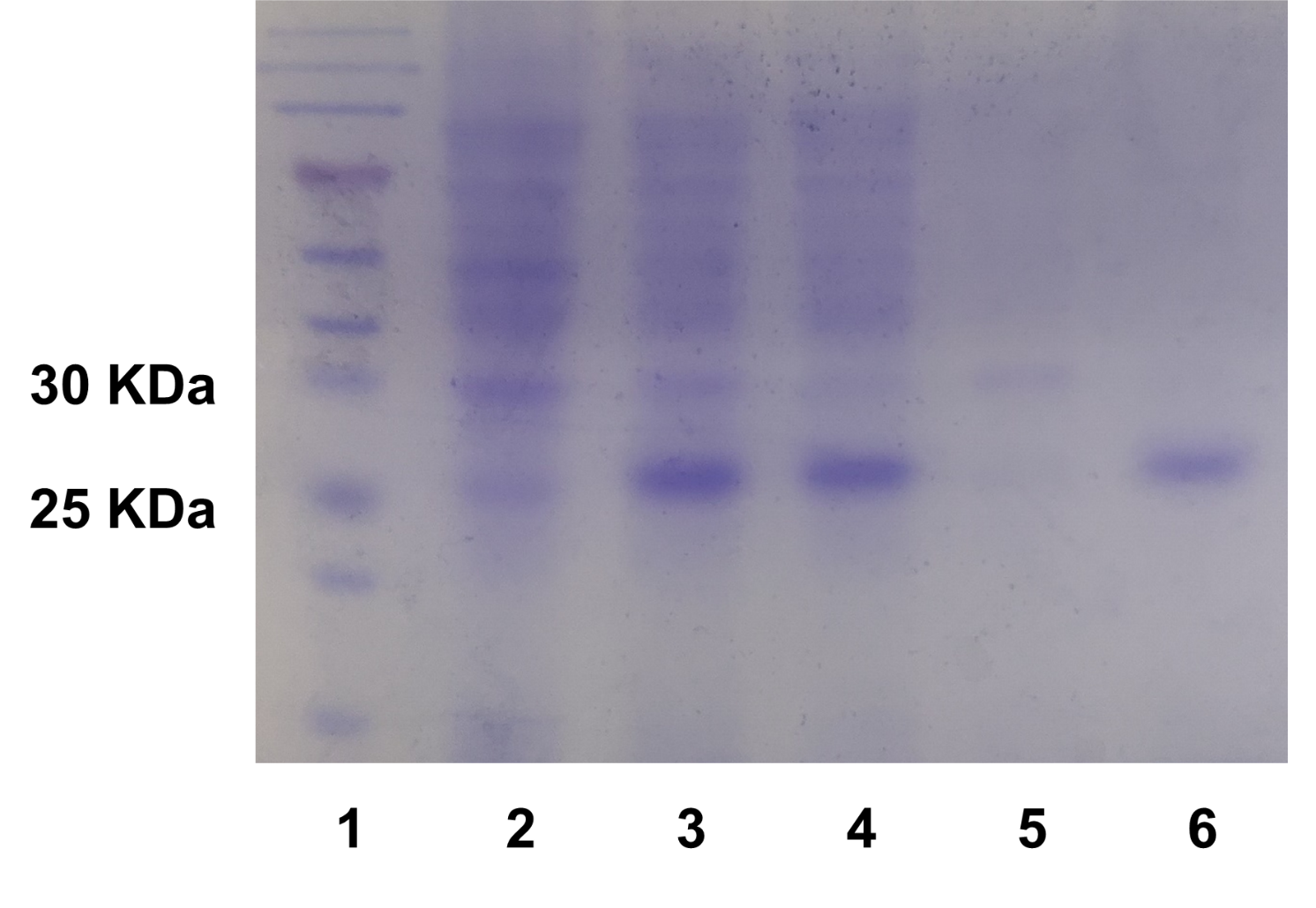


**Figure S2**. The recombinant expression of HFn protein. Lane 1, protein markers; Lane 2, the total protein of engineered bacteria before being induced by IPTG; Lane 3, the total protein of engineered bacteria after being induced by IPTG; Lane 4, the supernatant post ultrasonication; Lane 5, the sediment after ultrasonication, Lane 6, purified recombinant HFn.


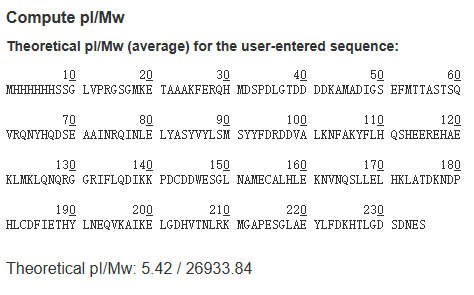


**Figure** **S3**. Theoretical pI/Mw of recombinant expressed HFn Predicted in Expasy (https://web.expasy.org/cgi-bin/compute_pi/pi_tool).

**Figure S4**. Fluorescent intensity from 360 nm to 450 nm excited by 280 nm of ferritin after mixing with Gallic acid at different ratio.


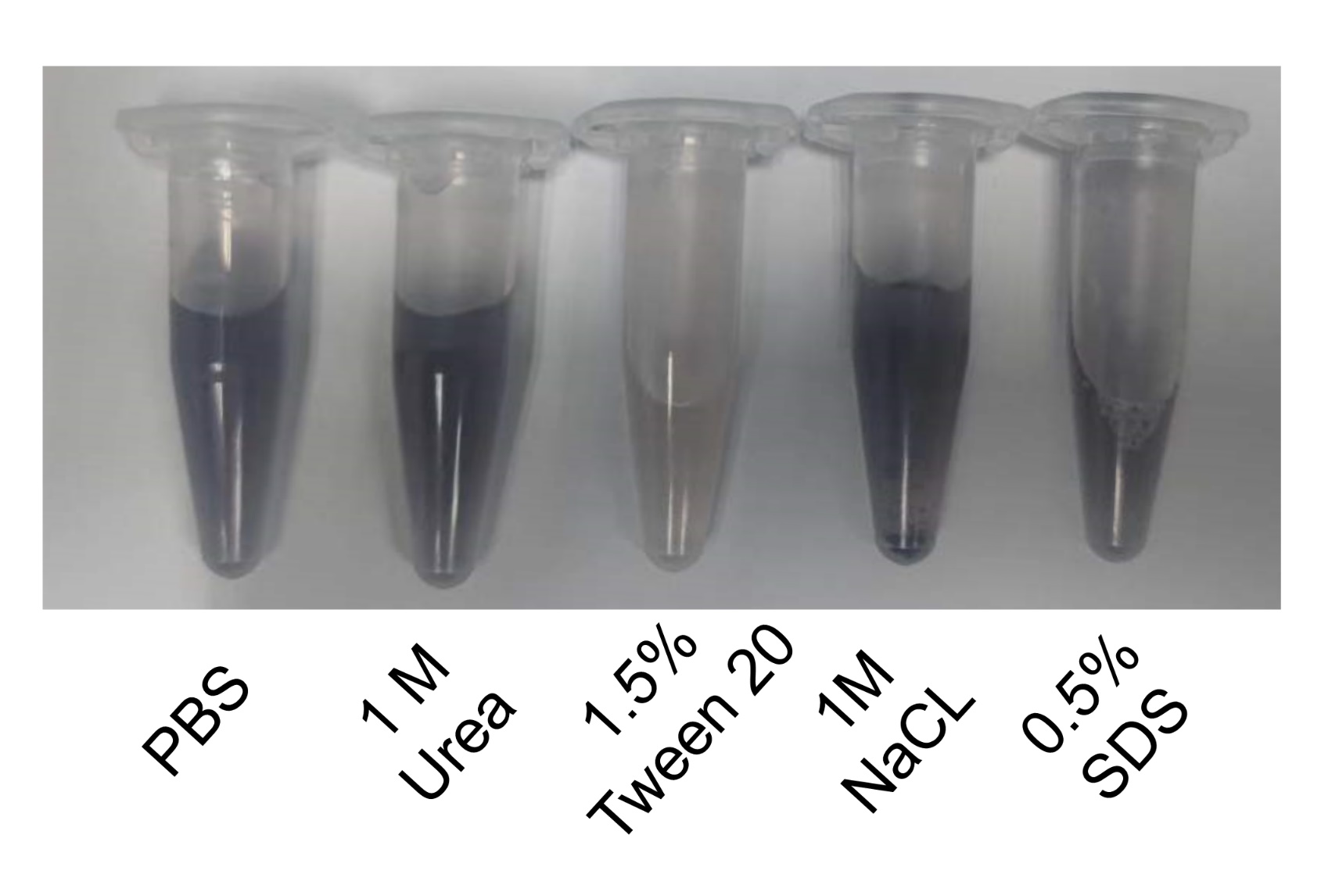


**Figure S5**. HFn/GA-Fe incubated in different solution for 24 h.


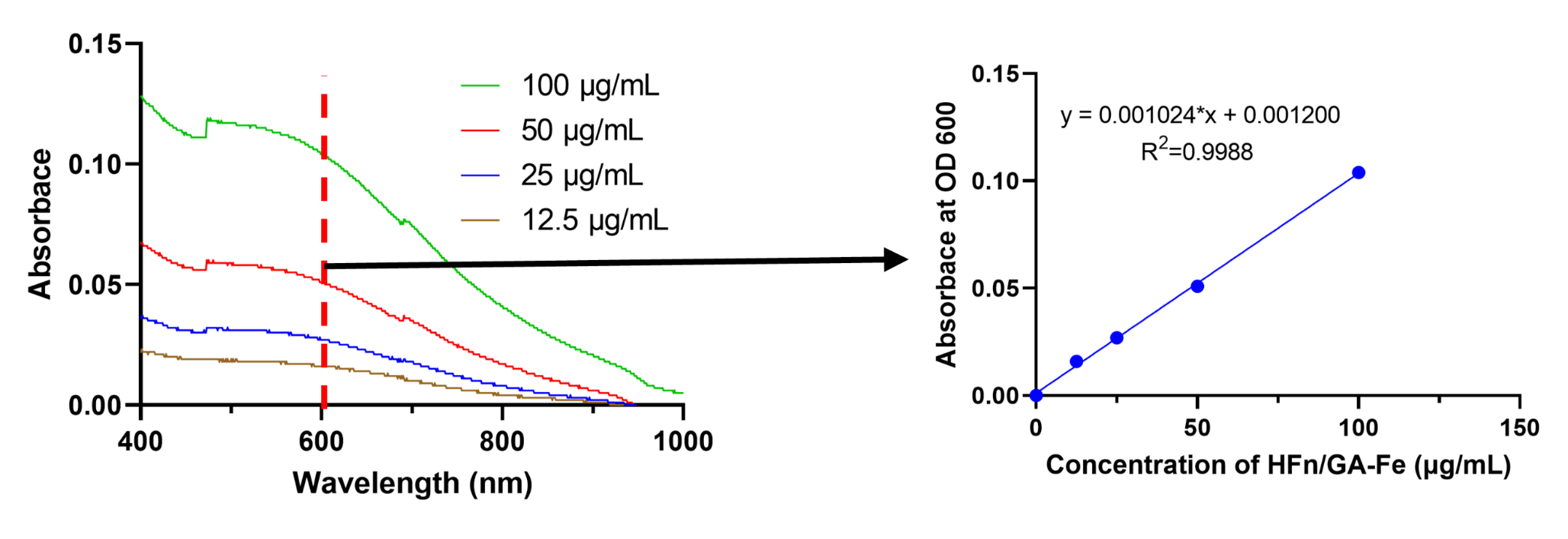


**Figure S6**. The curve fitting of OD600 value of different concentrations of HFn/GA-Fe nanoparticles.


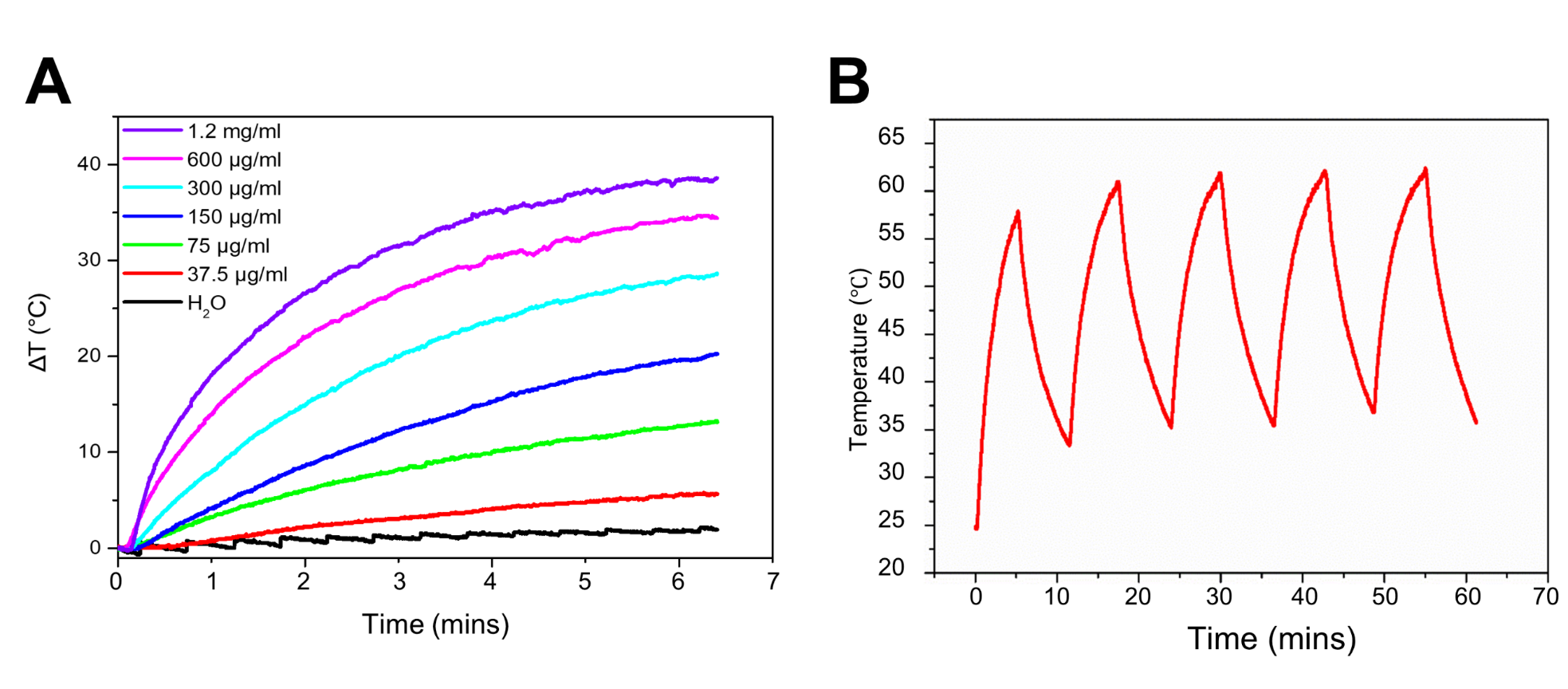


**Figure S7**. (A) Temperature increasing curve of HFn/GA-Fe at different concentrations. (B) Thermal-cycle test of HFn/GA-Fe.

**Figure** **S8**. Uptake curve of HFn/GA-Fe and GA-Fe by 4T1. Data are shown as mean ± SD (n = 3).


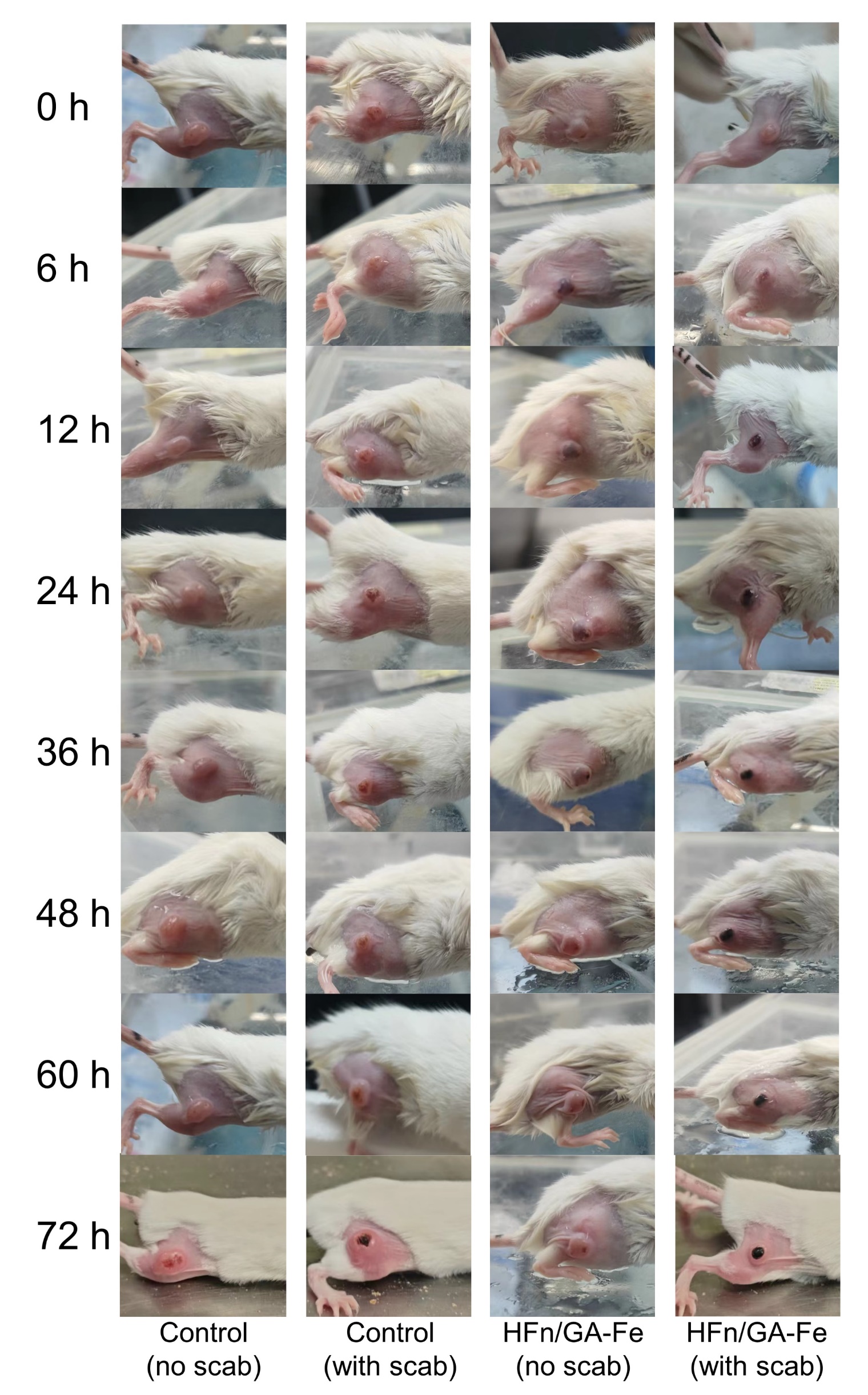


**Figure S9**. Representative photos of tumors at 0 h, 6 h, 12 h, 24 h, 36 h, 48 h, 60 h, and 72 h post-injection of PBS and HFn/GA-Fe nanoparticles to mice with/without scab.


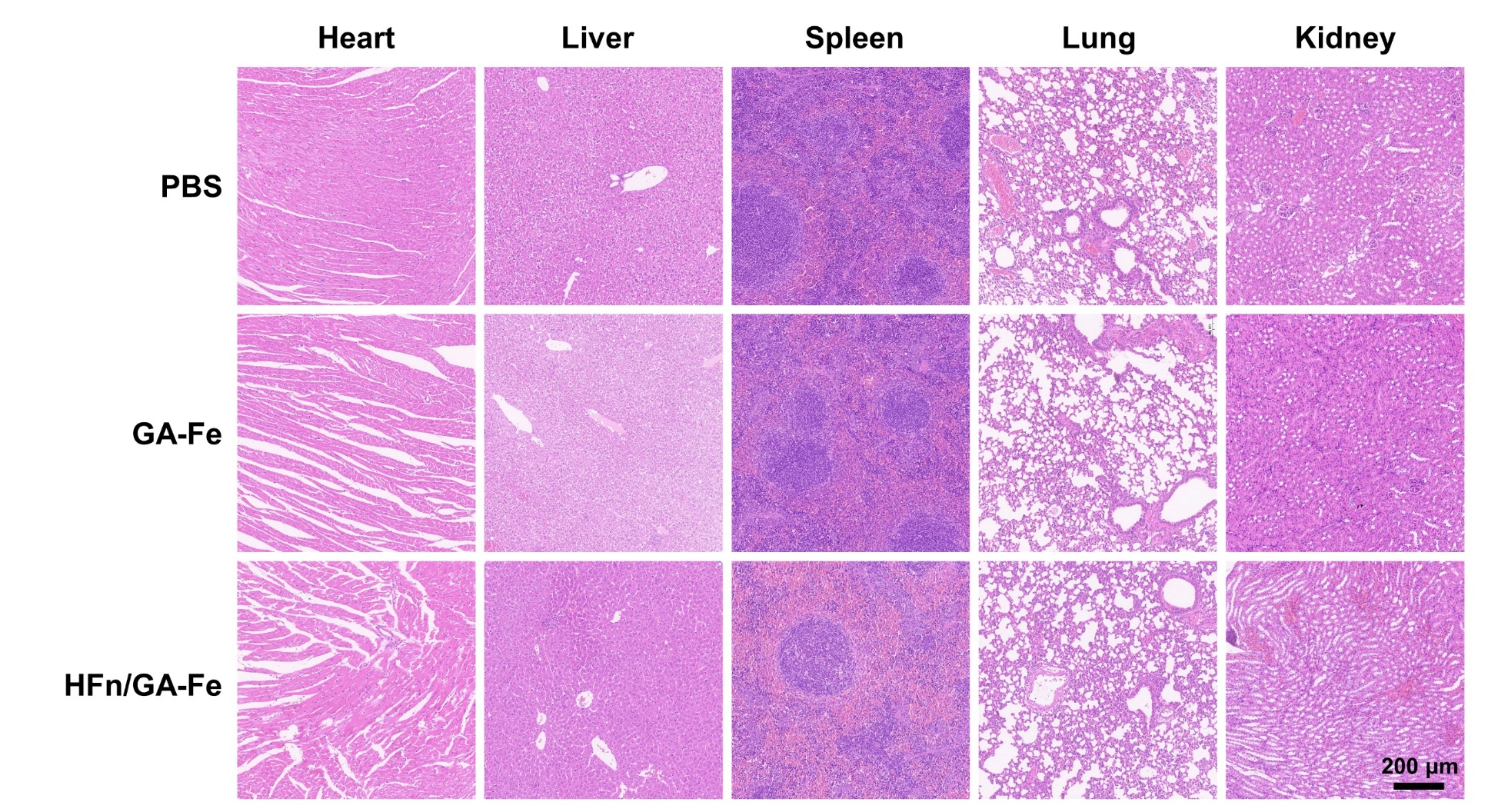


**Figure** **S10**. H&E stains of different organs at 12 h after intravenous injection of PBS, GA-Fe, and HFn/GA-Fe. Scale bars: 200 μm.


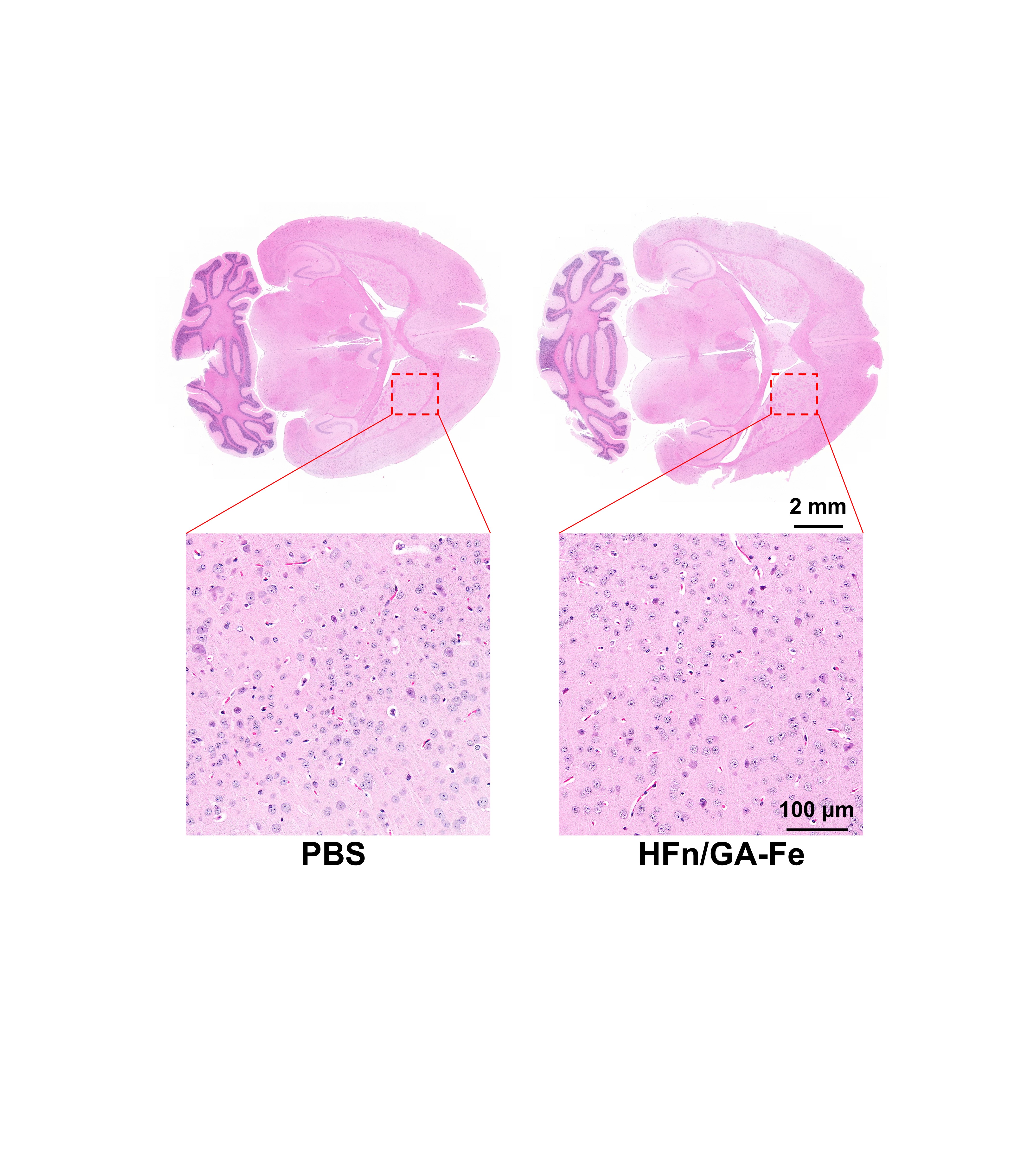


**Figure** **S11.** Whole H&E staining of mice brain at 12 h after intravenous injection of PBS and HFn/GA-Fe. Scale bars: 2 mm, 100 μm,

**Figure** **S12**. Iron element contents in different organs at 12h after intravenous injection of PBS, GA-Fe, and HFn/GA-Fe. Data are shown as mean ± SD (n = 5).


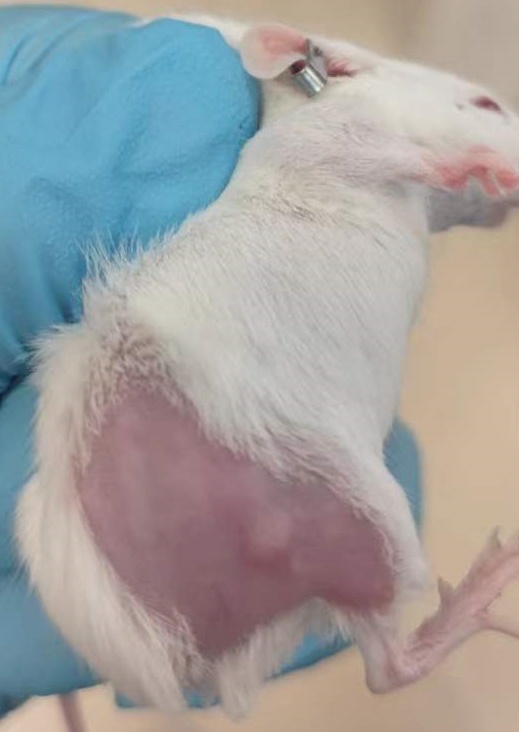


**Figure** **S13**. 4T1 tumor-beard mouse at 12 h after intravenously injected with free HFn.


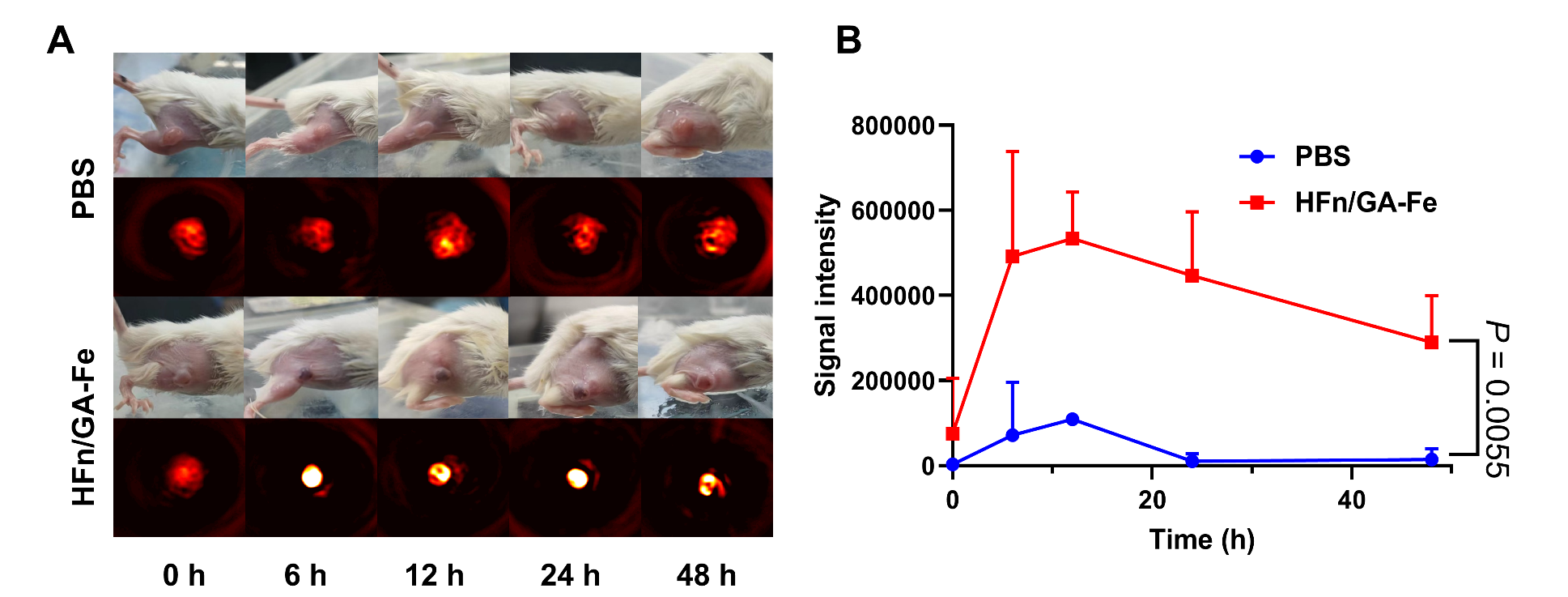


**Figure** **S1****4**. (A) Photoacoustic imaging and (B) relative signal intensity curve of tumor site at different time points post-injection of HFn/GA-Fe. Data are shown as mean ± SD (n = 3). All statistical analyses were performed using one-way ANOVA.


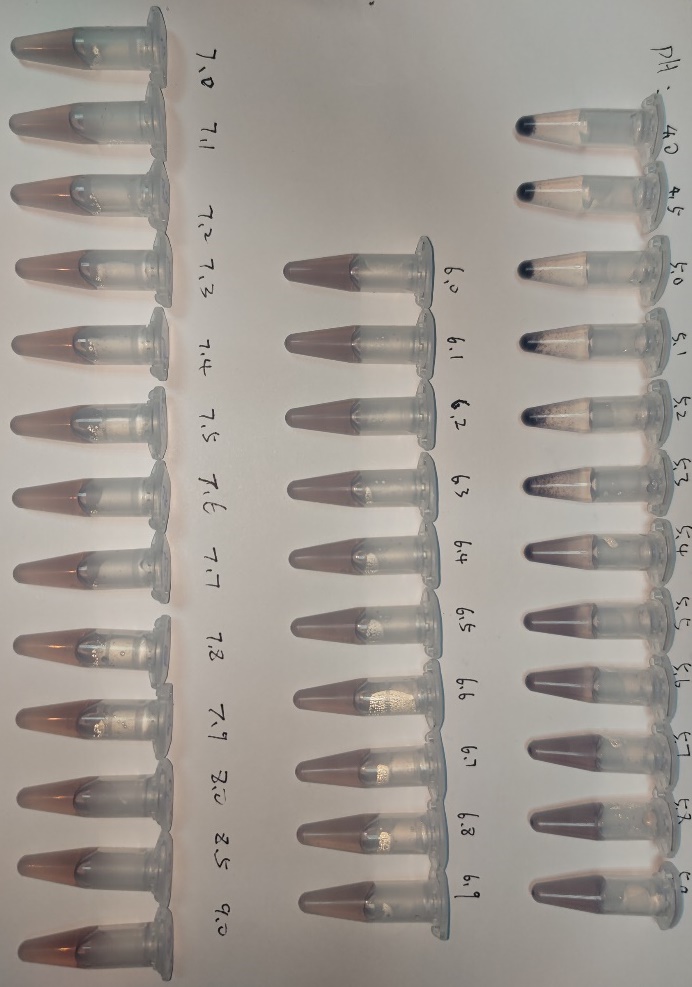


**Figure S15**. HFn/GA-Fe aggregation status under pH ranges from 4.0 to 9.0 at 24 h.


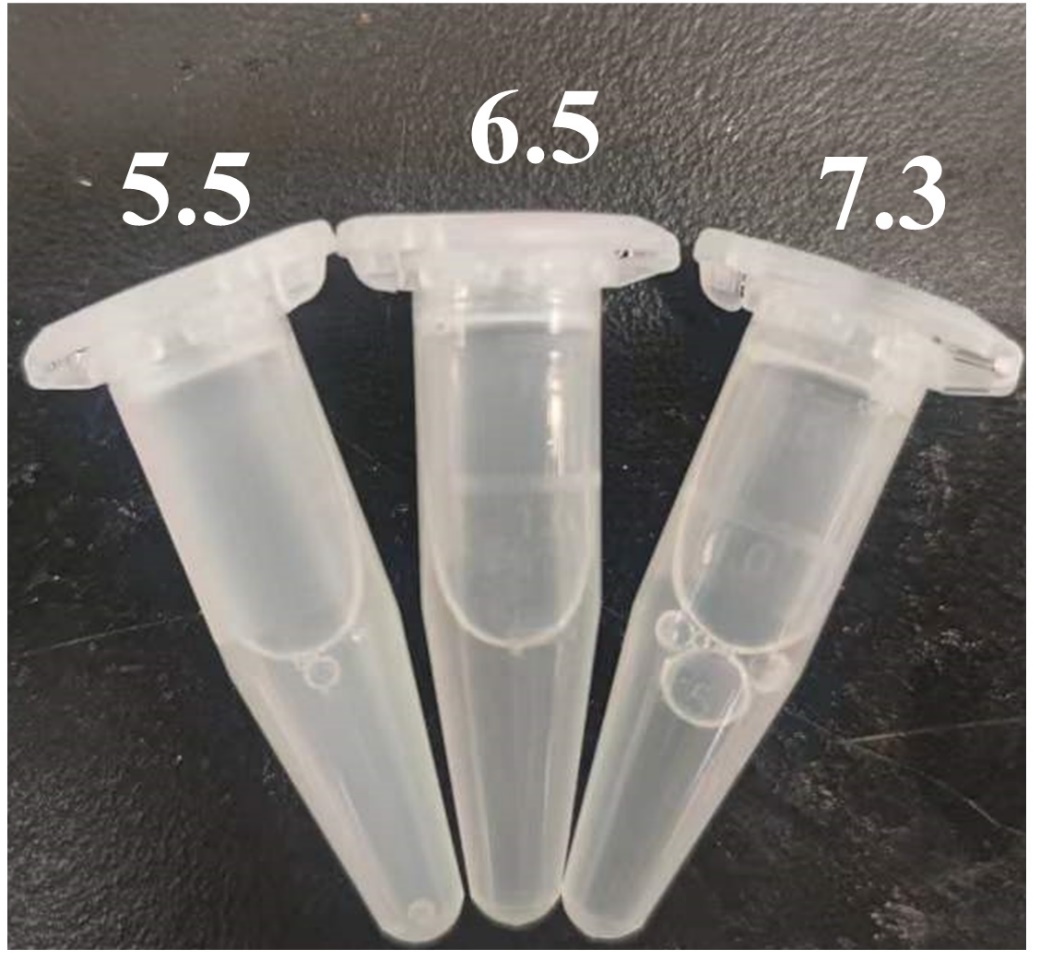


**Figure** **S16**. HFn aggregation status under pH 5.5, 6.5, and 7.3 at 2 h.

**Figure S17**. Mean Isoelectric point(pI) of ferritin heavy chain from different species.


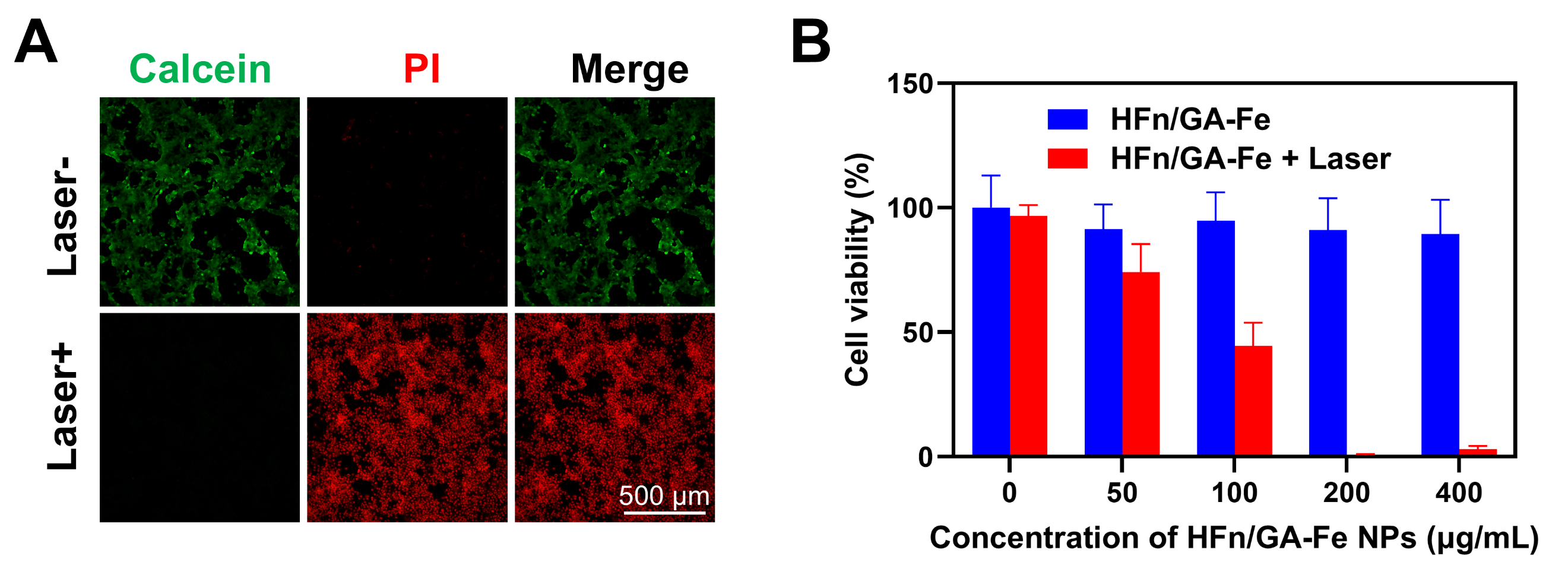


**Figure** **S18**. Photothermal therapy *in vitro*. (A) Live and dead staining of 4T1 at 6 h after photothermal therapy. Scale bars: 500 μm. (B) The viability of 4T1 cells posts photothermal therapy at different concentrations of HFn/GA-Fe. Data are shown as mean ± SD (n = 6).


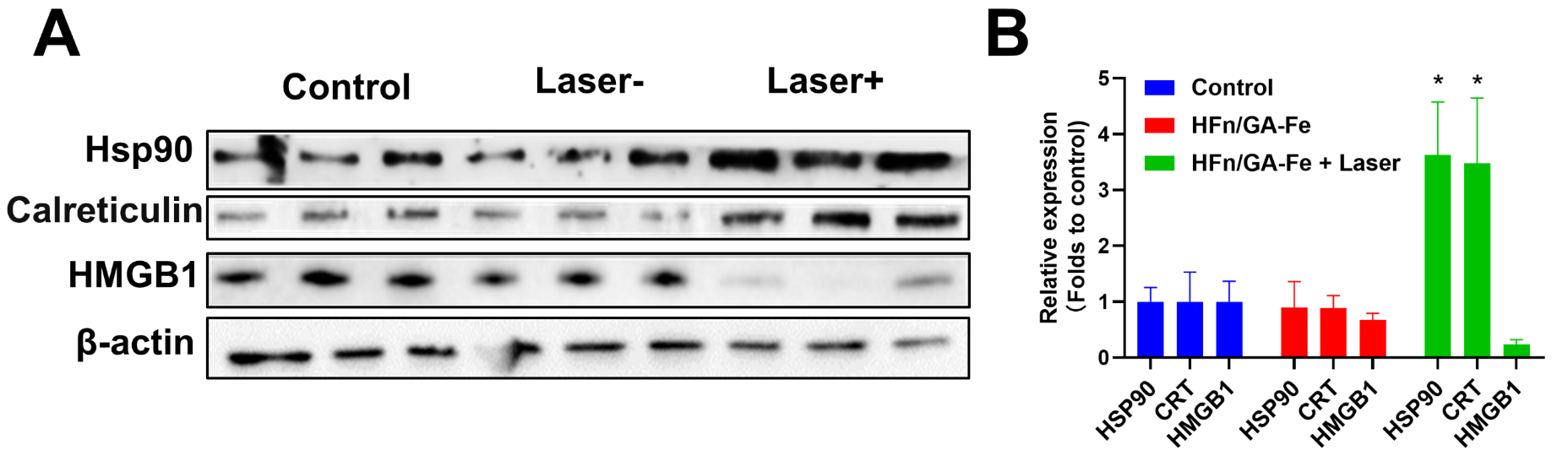


**Figure** **S19**. (A) Content analysis and (B) grayscale quantification of ICD markers including CRT, HMGB1, and Calreticulin by western blot. Data are shown as mean ± SD (n = 3).


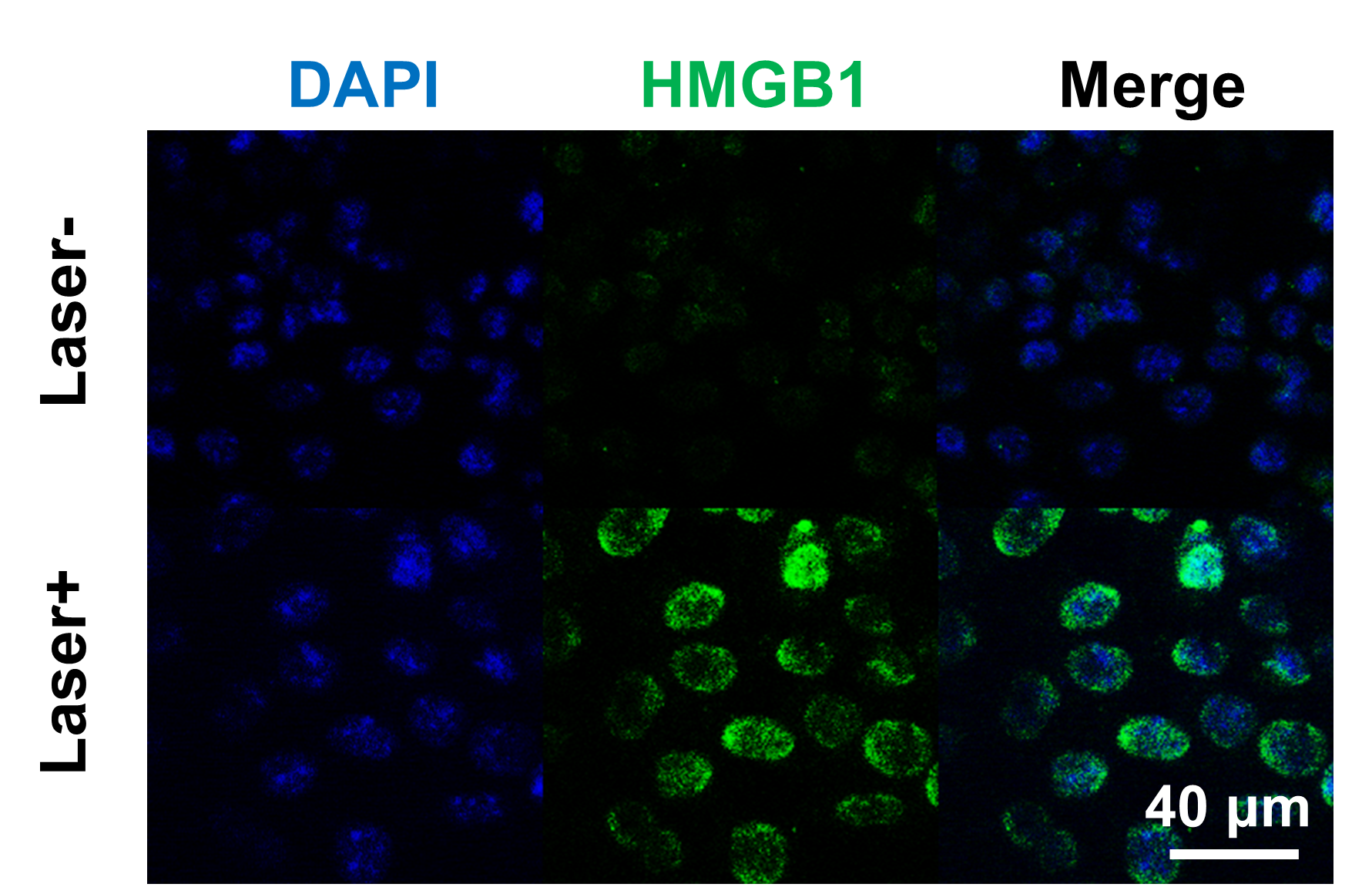


**Figure** **S20**. Immunofluorescence of HMGB1 before and post laser irradiation. Scale bars: 40 μm.


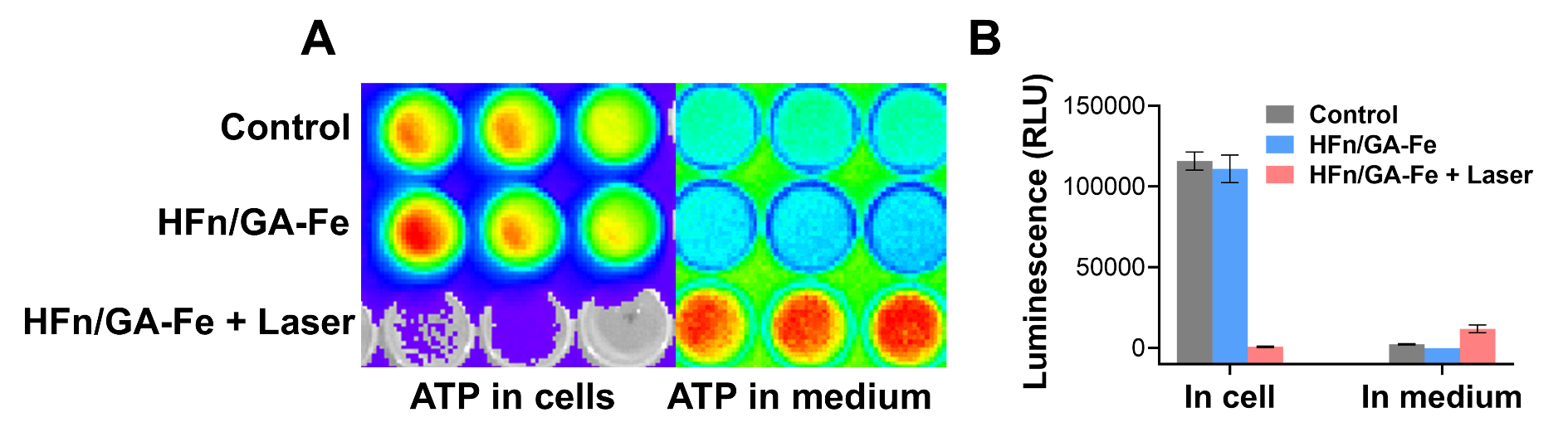


**Figure** **S21**. (A-B) The imaging and statistics of ATP concentration in cells and in the medium. Data are shown as mean ± SD (n = 3).


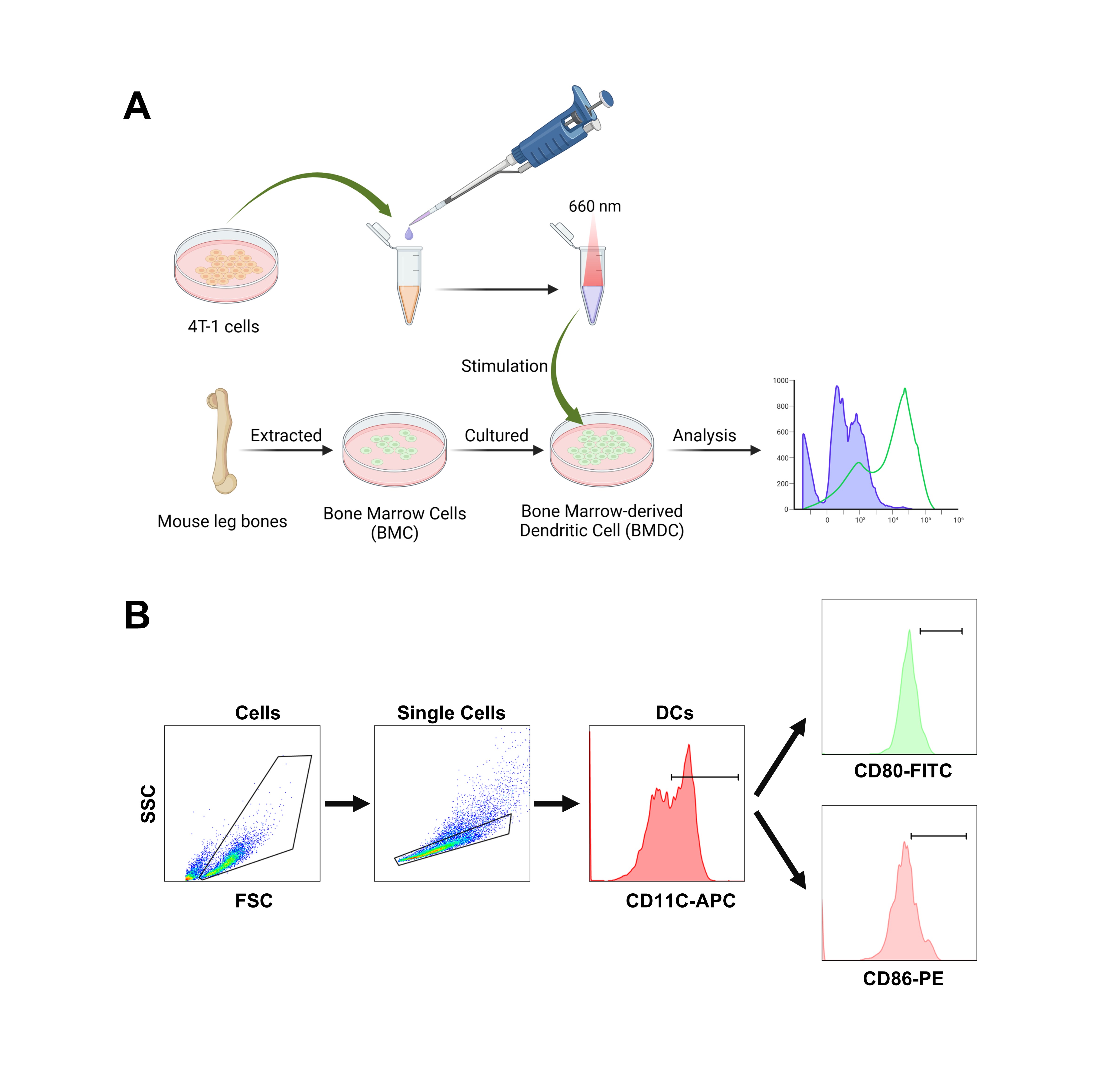


**Figure** **S22**. Maturation of DC cells *in vitro*. (A) Schematic diagram of DCs maturation experiments. (B) Gating strategy of the CD80 and CD86 on DCs.


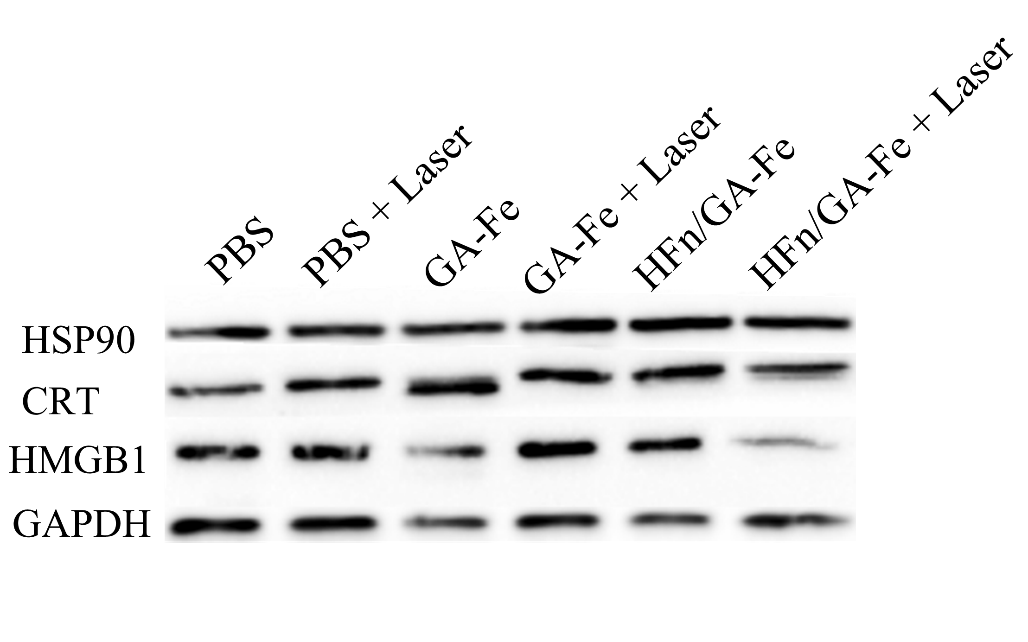


**Figure** **S23**. Content analysis of ICD markers including HSP90, CRT, and HMGB1 of 4T1 tumor by western blot.


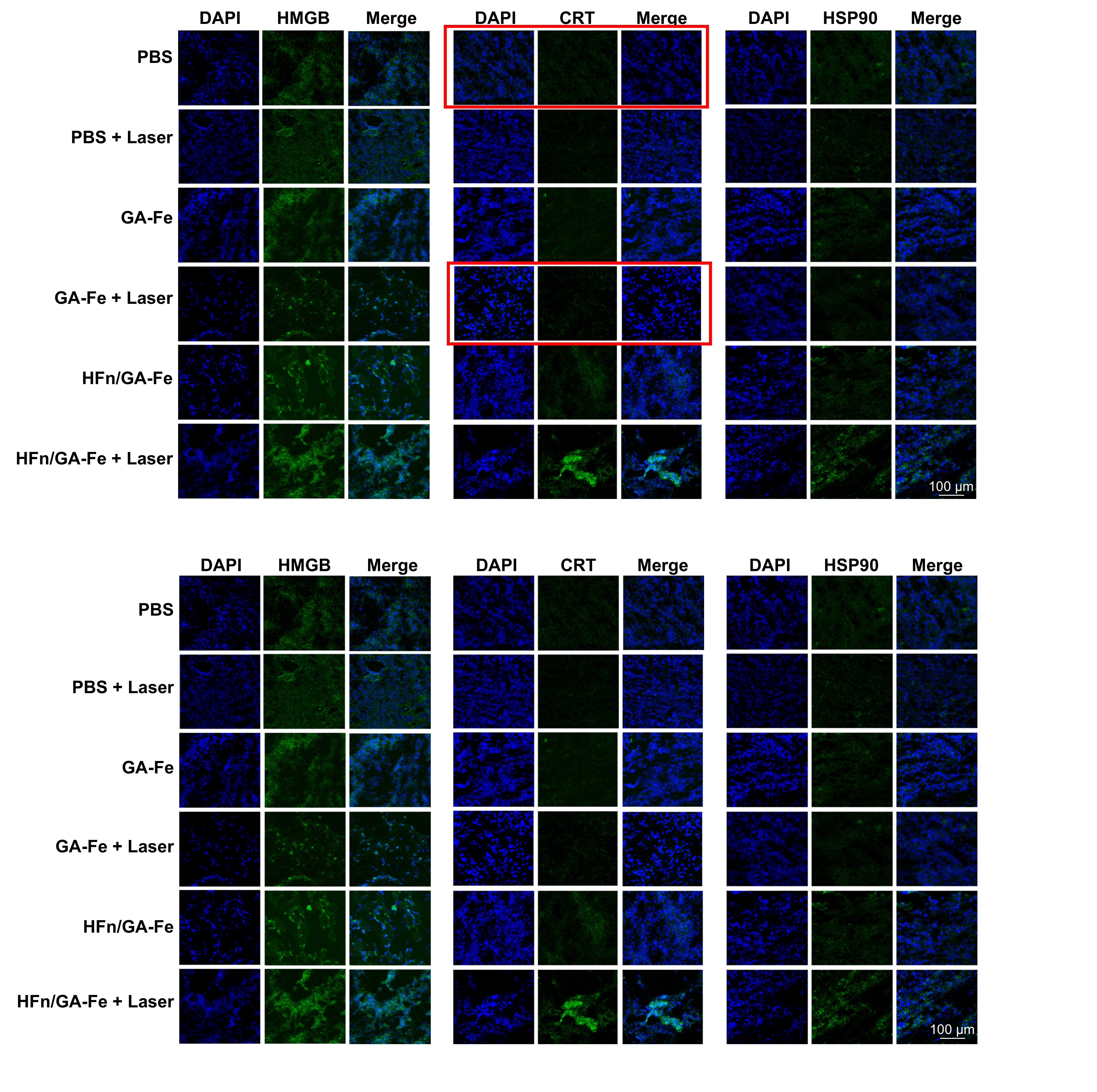


**Figure** **S24**. Immunofluorescence of HMGB1, CRT, and HSP90 of 4T1 tumor in different groups including PBS, PBS + Laser, GA-Fe, GA-Fe + Laser, HFn/GA-Fe, HFn/GA-Fe + Laser. Scale bars: 100 μm.


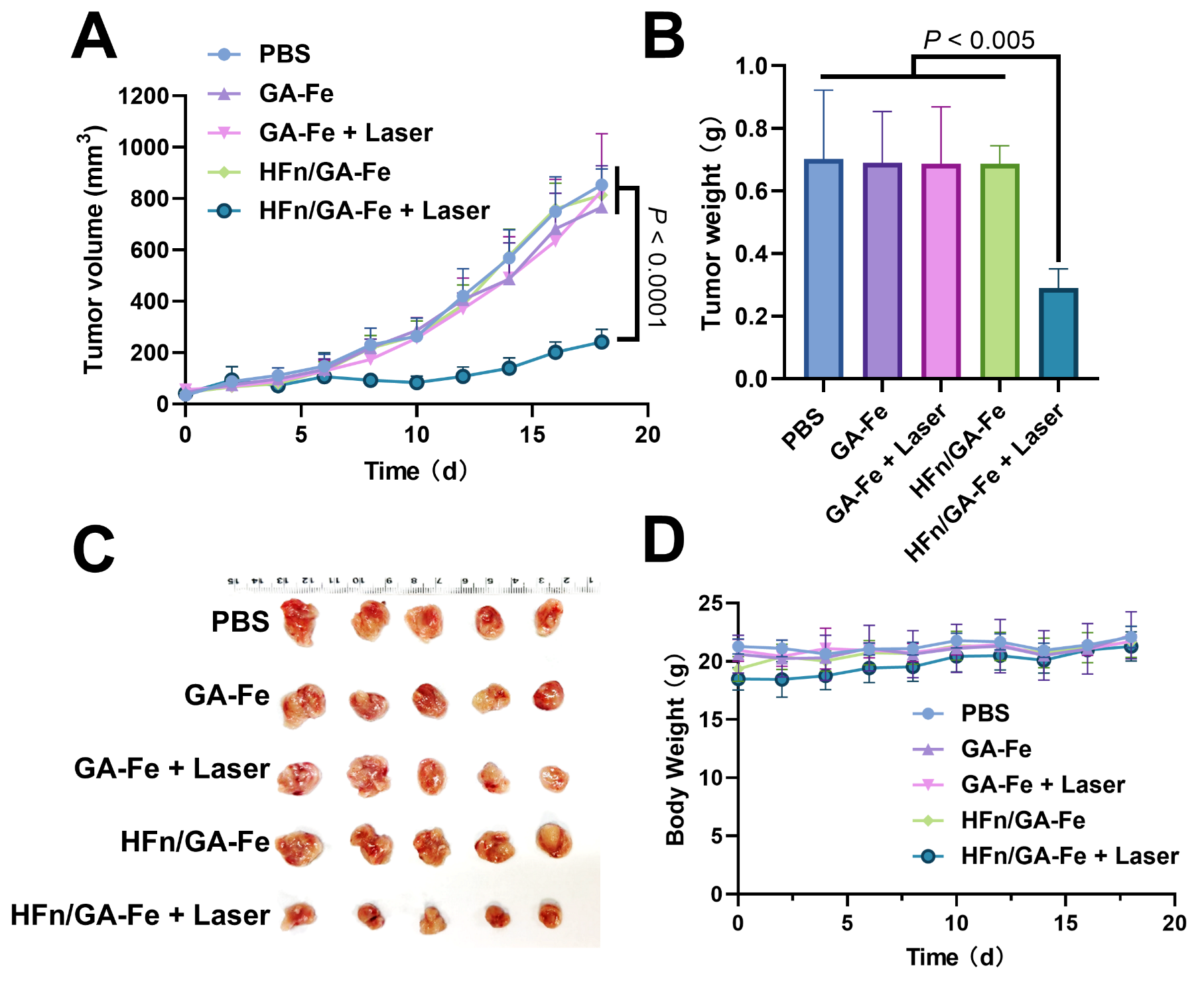


**Figure** **S25**. (B) Tumor volume growth curve after treating with PBS, GA-Fe, GA-Fe + laser, HFn/GA-Fe, and HFn/GA-Fe + laser. (C) Average tumor weight in different groups. (D) Tumor picture of different groups. (F) Mouse weight of different groups. Data are shown as mean ± SD (n = 5). All statistical analyses were performed using one-way ANOVA.


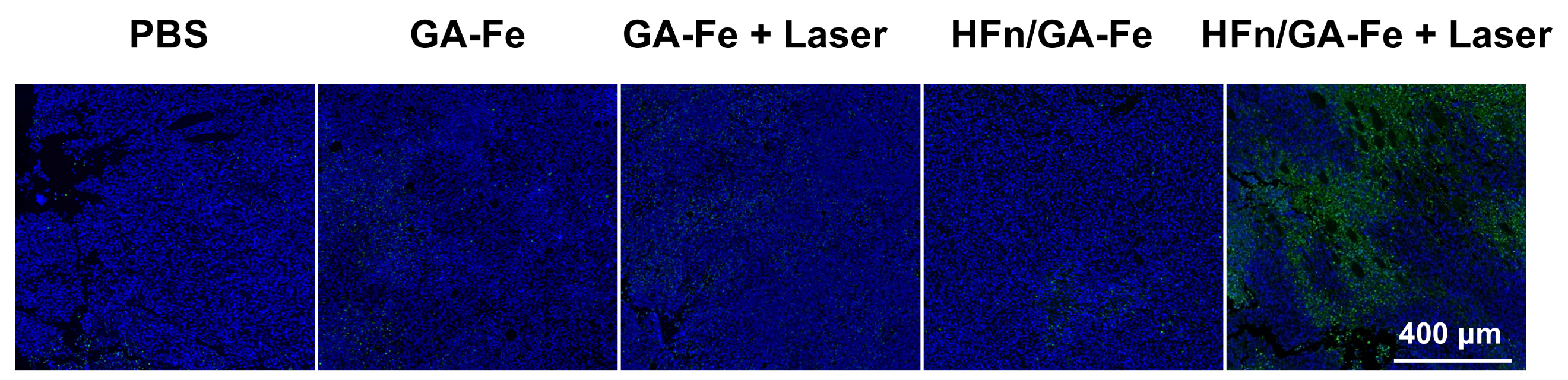


**Figure** **S26**. TUNEL staining of different groups in animal experiment. Scale bars: 400 μm.


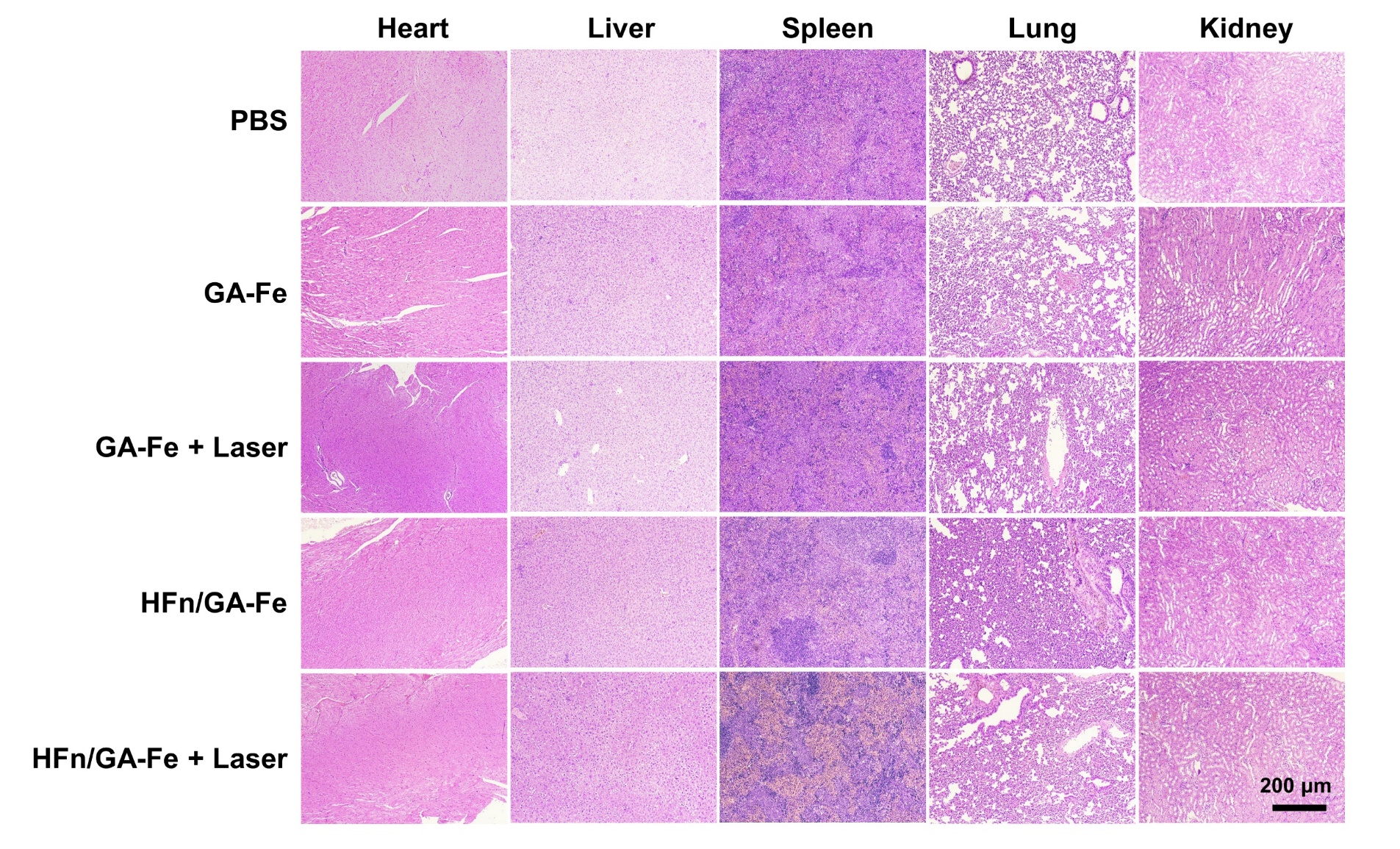


**Figure** **S27**. H&E stains of different organs after photothermal therapy. Scale bars: 200 μm.


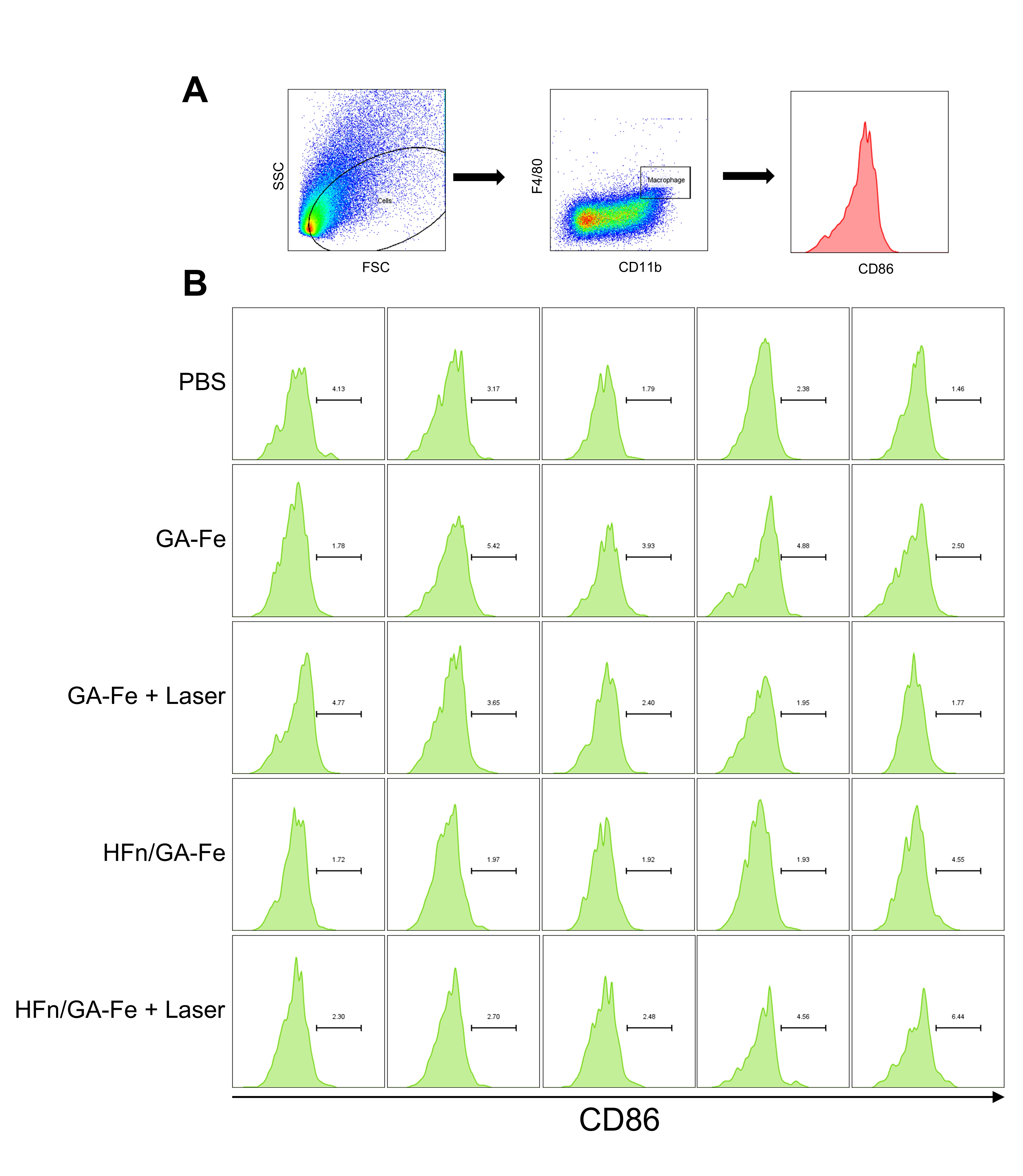


**Figure** **S28**. (A) Gating strategy of the CD86 expression of macrophage. (B) Flow-cytometry analysis of CD86 expression of macrophage in 4T1 tumor in different groups.


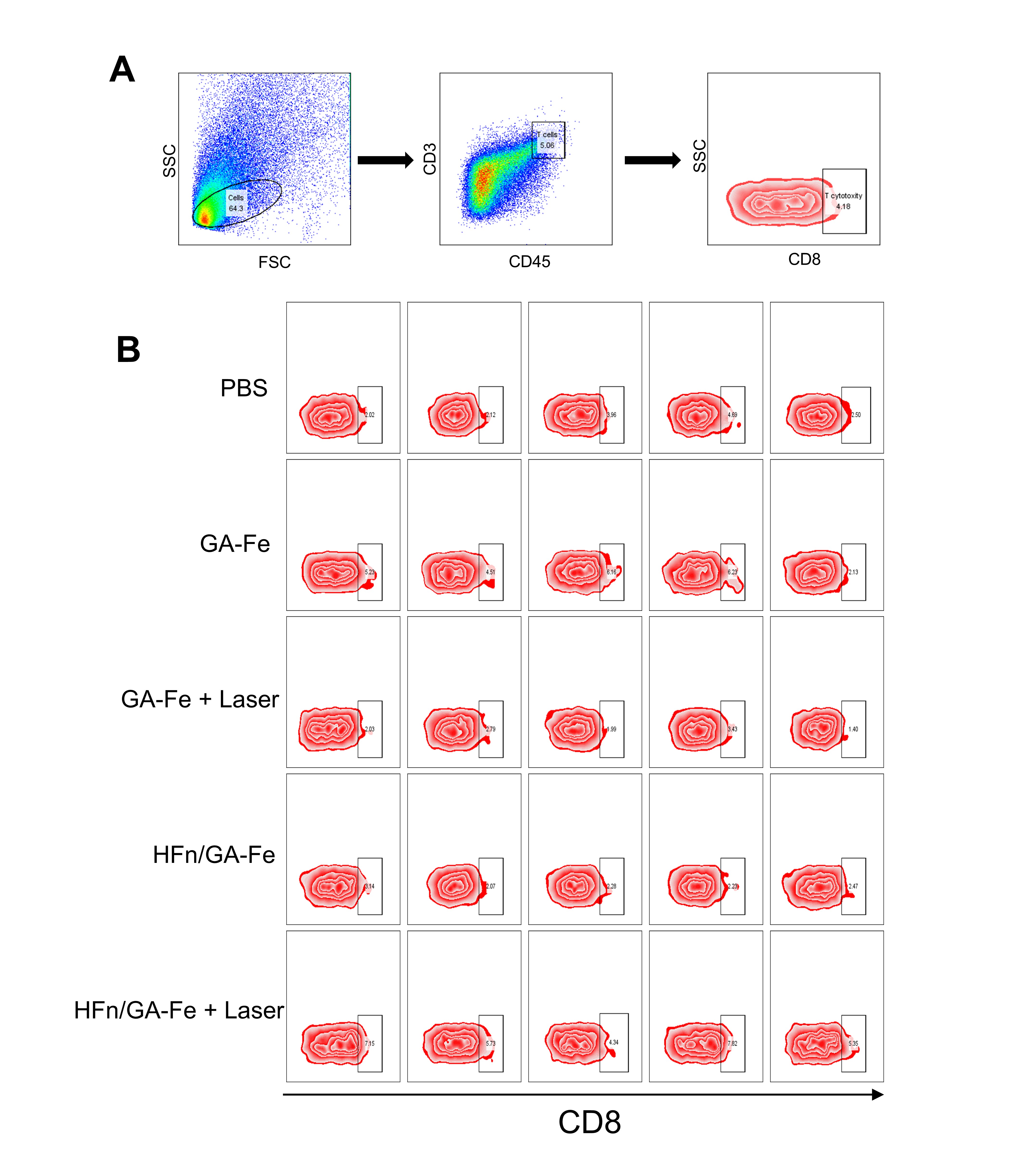


**Figure S29**. (A) Gating strategy of the CD8+ T cells. (B) Flow-cytometry analysis of CD8+ T cells in 4T1 tumor in different groups.

**Figure** **S30**. The fitting of fluorescent intensity of different concentrations of Dox (Ex: 497 nm, Em: 555 nm).


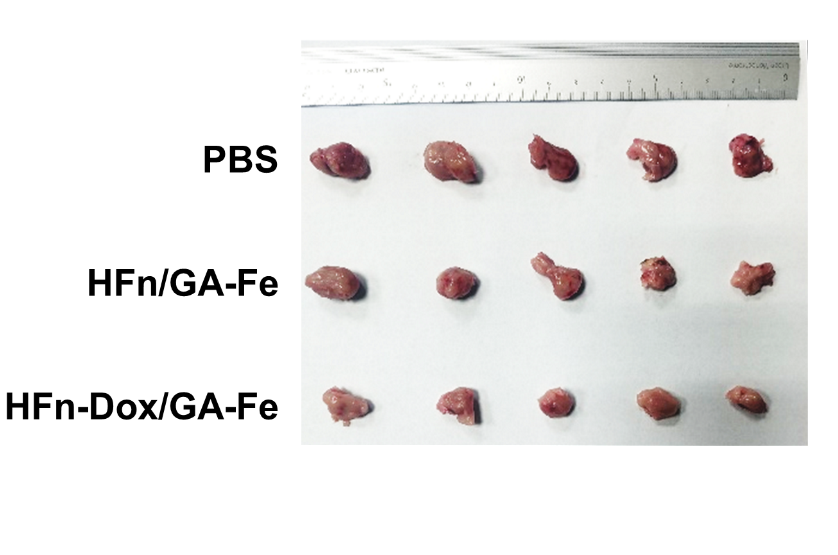


**Figure** **S31**. Tumor picture of different groups


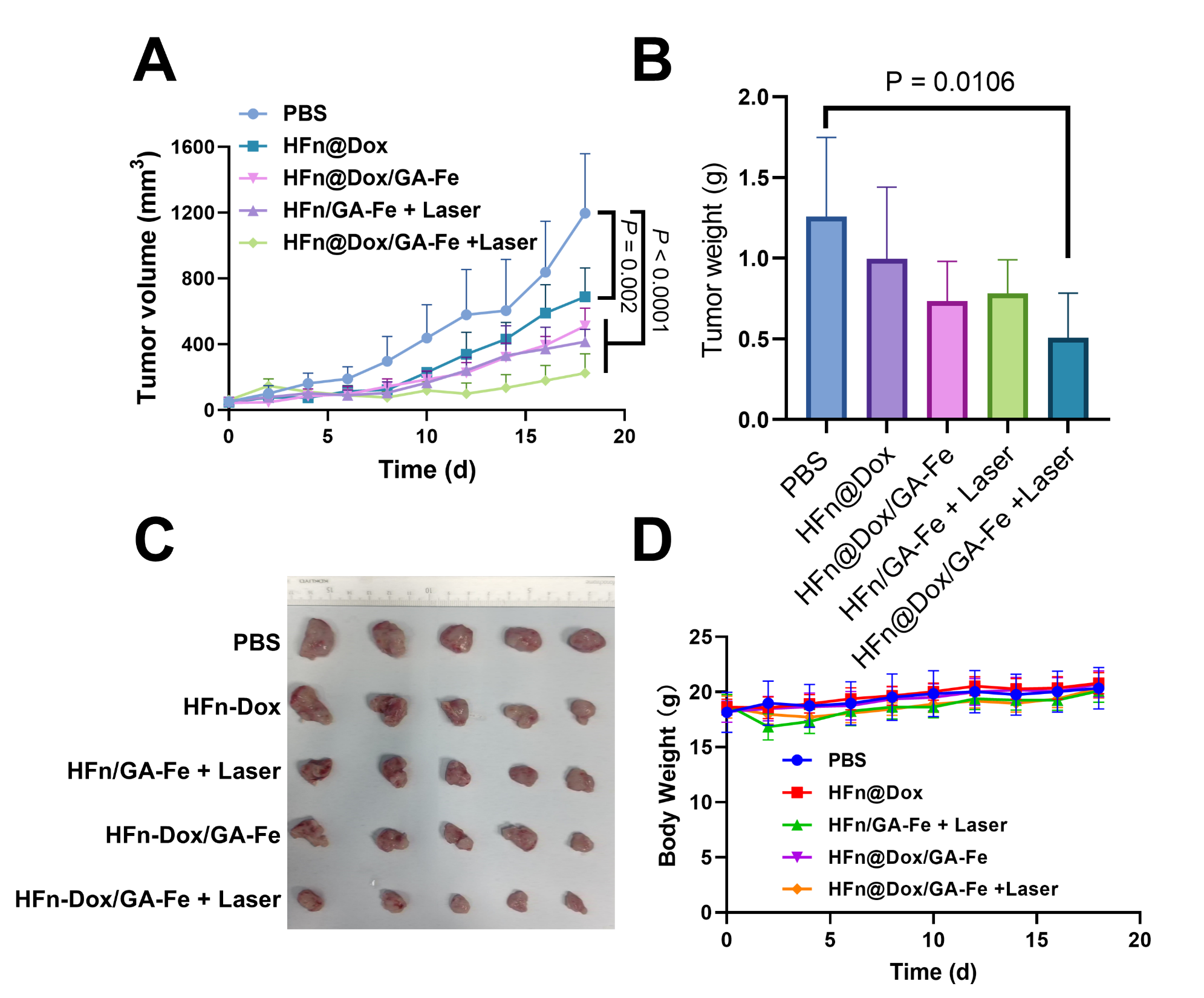


**Figure** **S32**. (A) Average tumor volume growth curve after treatment. (B) Tumor weight of different groups. (C) Tumor picture of different groups. (D) Mouse weight of different groups. Data represents mean values and SD for n=5. Data are shown as mean ± SD (n = 5). All statistical analyses were performed using one-way ANOVA.

**Table S1**. Isoelectric point(pI) of ferritin heavy chain from different species.

| Species | Theoretical pI | Homology |
| --- | --- | --- |
| *Homo sapiens* | 5.31 | 100.00% |
| *Rattus norvegicus* | 5.62 | 93.96% |
| *Mus musculus* | 5.53 | 92.31% |
| *Bos taurus* | 5.54 | 91.16% |
| *Felis catus* | 5.53 | 95.08% |
| *Anser cygnoides* | 5.74 | 91.21% |
| *Chionodraco rastrospinosus* | 5.34 | 71.66% |
| *Xenopus laevis* | 5.61 | 68.18% |
| *Pongo abelii* | 5.31 | 99.45% |

**Table S2**. Complete blood count of short-term (S1-S3) at 12 h post one treatment and long-term (L1-L3) on eight days after four treatments with HFn/GA-Fe.

| Parameter | S1 Value | S2 Value | S3 Value | L1 Value | L2 Value | L3 Value | Unit | Reference range |
| --- | --- | --- | --- | --- | --- | --- | --- | --- |
| WBC | 5.9 | 5.3 | 2.9 | 8.0 | 7.7 | 7.6 | 10^9/L | 0.8-10.6 |
| Lymph# | 4.5 | 4.1 | 2.2 | 6.3 | 6.4 | 6.1 | 10^9/L | 0.6-8.9 |
| Mon# | 0.2 | 0.1 | 0.0 | 0.3 | 0.2 | 0.2 | 10^9/L | 0.04-1.4 |
| Gran# | 1.2 | 1.1 | 0.7 | 1.4 | 1.1 | 1.3 | 10^9/L | 0.23-3.6 |
| Lymph% | 77.0 | 77.2 | 75.3 | 79.2 | 83.2 | 79.7 | % | 40-92 |
| Mon% | 2.9 | 2.4 | 2.1 | 3.2 | 2.5 | 2.9 | % | 0.9-18 |
| Gran% | 20.1 | 20.4 | 22.6 | 17.6 | 14.3 | 17.4 | % | 6.5-50 |
| RBC | 10.37 | 9.50 | 9.06 | 8.92 | 8.37 | 9.23 | 10^12/L | 6.5-11.5 |
| HGB | 167 | 160 | 152 | 151 | 139 | 142 | g/L | 110-165 |
| HCT | 50.2 | 47.5 | 42.9 | 43.5 | 37.6 | 42.4 | % | 35-55 |
| MCV | 48.5 | 50.0 | 47.4 | 48.8 | 45.0 | 46.0 | fL | 41-55 |
| MCH | 16.1 | 16.8 | 16.7 | 16.9 | 16.6 | 15.3 | pg | 13-18 |
| MCHC | 332 | 336 | 354 | 347 | 369 | 334 | g/L | 300-360 |
| RDW | 14.4 | 14.1 | 17.0 | 14.4 | 17.3 | 14.3 | % | 12-19 |
| PLT | 1026 | 1217 | 951 | 836 | 606 | 866 | 10^9/L | 400-1600 |
| MPV | 7.2 | 6.9 | 6.3 | 6.7 | 6.3 | 6.4 | fL | 4.0-6.2 |
| PDW | 17.4 | 17.0 | 17.0 | 16.9 | 16.8 | 16.6 |  | 12.0-17.5 |
